# PP2A-dependent downregulation of GABA_B_ receptors modulates somatostatin interneurons function and plasticity

**DOI:** 10.1016/j.isci.2026.117878

**Published:** 2026-10-27

**Authors:** Nithya Sethumadhavan, Max A. Wilson, Anna Sumera, Desiree Loreth, Rita M. Loureiro, Imre Vida, Akos Kulik, Sam A. Booker

**Affiliations:** 1 Institute of Physiology II, Faculty of Medicine, University of Freiburg, Freiburg, Germany; 2 Institute for Cardiovascular and Neuroscience Research, University of Edinburgh, Edinburgh, UK; 3 Simons Initiative for the Developing Brain, University of Edinburgh, Edinburgh, UK; 4 Patrick Wild Centre for Autism Research, University of Edinburgh, Edinburgh, UK; 5 Charité Universitätmedizin, Berlin, Germany; 6 Institute for Biology, Humboldt – Universität zu Berlin, Berlin, Germany

**Keywords:** GABAB receptor, somatostatin interneuron, long-term potentiation, protein phosphatase 2A, electron microscopy, electrophysiology

## Abstract

Cortical circuits require precise inhibitory and excitatory neurotransmission to encode information and prevent pathology. Metabotropic GABA_B_ receptors (GABA_B_Rs) are key inhibitory regulators in mammalian neurons. In GABAergic interneurons (INs), GABA_B_R activation reduces inhibition, leading to disinhibitory mechanisms. Hippocampal somatostatin-expressing (SST) INs provide feedback inhibition to distal dendrites of principal cells (PCs) and other INs. Plasticity of SST INs is well established to control hippocampal circuit function dependent on metabotropic glutamate receptors (mGluRs) and GABA_B_Rs. Whether GABA_B_Rs regulate SST INs function over longer time scales and if this influences circuit function remains unclear.

We combine quantitative freeze-fracture replica immunoelectron microscopy, *ex vivo* electrophysiology, and pharmacology to show that GABA_B_R activation regulates long-term potentiation (LTP) of SST INs through protein phosphatase 2A (PP2A)-dependent loss of cell-surface GABA_B_R, mGluR1α receptors, and Ca_v_1.2 (L-type) Ca^2+^ channels. GABA_B_R loss in SST INs shifts hippocampal activity toward greater plasticity of long-range inputs to CA1.

## Highlights

• GABA_B_ receptor activation leads to its own loss from somatostatin neuron membranes
• Long-term potentiation is impaired via loss of calcium channels
• Inhibiting protein phosphatase 2A, which prevent receptor and channel loss
• GABA_B_ receptor activation leads to long-term alteration to circuit-level plasticity

## Graphical abstract

**Figure:**
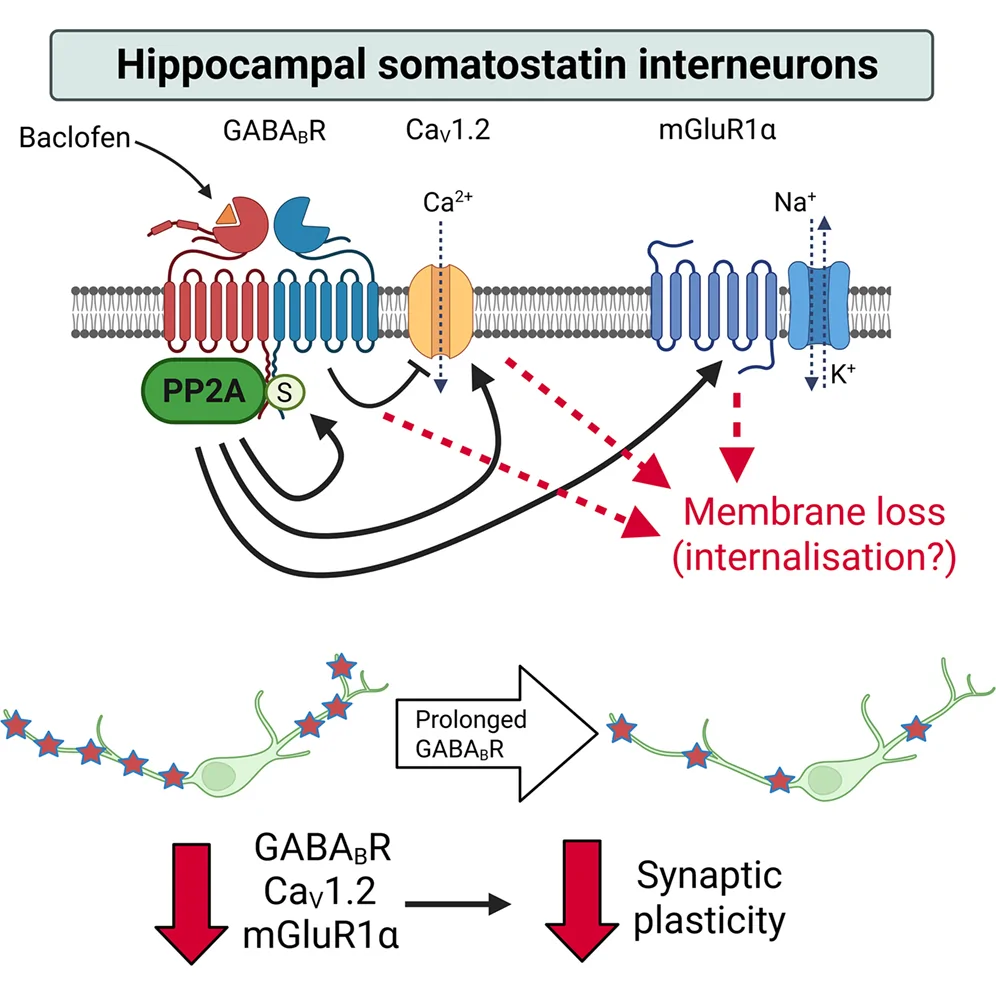

## Teaser

Molecular network; Cellular neuroscience; Sensory neuroscience; Cell biology

## Introduction

The appropriate function of cortical microcircuits relies on dynamically balanced excitatory and inhibitory synaptic transmission, which maintains the neural code and contributes to memory formation.^1^^,^^2^^,^^3^ How these circuits respond to changing activity levels, or sustained pharmacological activity, is crucial to understanding how the brain functions at rest and how neuropathological conditions lead to altered behavior and cognition.

Synaptic inhibition is produced, in large part, by a heterogenous population of local interneurons (INs). These INs release GABA from their presynaptic terminals, which activates both synaptic and extrasynaptic ionotropic GABA_A_ receptors (GABA_A_Rs) and metabotropic GABA_B_ receptors (GABA_B_Rs), on excitatory principal cells and INs alike.^4^ Such inhibition of INs reduces their activity, leading to reduced local GABA release, and thus a reduction of inhibitory control—known as disinhibition. Dynamic interactions between inhibition and disinhibition are critical to ensure the timing and strength of neuronal activity leading to complex behavior. To date, most studied forms of disinhibition focus on direct synaptic GABA_A_R-mediated effects, which can regulate circuit level plasticity in hippocampus^5^ and neocortex.^6^ While the direct effect of GABA_B_R signaling on INs has been well described by ourselves and others,^7^^,^^8^^,^^9^^,^^10^^,^^11^^,^^12^^,^^13^^,^^14^ how their activation shapes the long-term excitability of inhibitory cells and influence circuit activity remains less well understood.

In CA1 of the hippocampus, a major type of IN are those which express the neuropeptide somatostatin (SST),^15^ which have their somatodendritic domain within *stratum oriens/alveus* (*str*. O/A) receiving inputs from local PCs,^10^^,^^16^^,^^17^ with axons ramifying in *str. lacunosum-moleculare* (*Str. L-M*). Thus, SST INs provide powerful feedback inhibition into the CA1 circuit,^18^ leading to complex circuit control. First, they directly inhibit distal dendrites of CA1 PCs co-terminating with entorhinal cortex inputs to strongly inhibit spatial information.^19^^,^^20^^,^^21^ Second, SST INs disinhibit proximal dendritic domains and thus gate contextual inputs from CA3.^22^ As such, associative long-term potentiation (LTP) of CA1 PC inputs to SST INs leads to a functional re-arrangement of the hippocampal information transfer, favoring CA3 inputs^22^^,^^23^ and are potentially able to alter the circuit dynamics of the hippocampus over behaviorally relevant timescales.^24^ We have shown previously that GABA_B_Rs acutely inhibit the induction of LTP from local CA1 PCs onto SST IN dendrites in *str. O/A*, via direct inhibition of Ca_v_1.2 containing L-type voltage-gated Ca^2+^ channels^11^ (VGCCs). Beyond these effects on LTP induction, GABA_B_Rs also strongly regulate local circuit integration of SST INs at both synaptic inputs from, and their outputs to, CA1 PCs.^10^ Given the high affinity of GABA for GABA_B_Rs and their plethora of accessory proteins, they undergo rapid desensitization^8^^,^^25^^,^^26^^,^^27^^,^^28^^,^^29^ and agonist-dependent acceleration of internalization.^30^^,^^31^ Furthermore, GABA_B_Rs are known to interact with metabotropic glutamate receptors (mGluRs), preferentially with type 1α receptors (mGluR1α), as shown in the cerebellum.^32^^,^^33^ Considering the high density of both mGluR1α and GABA_B_Rs on SST INs^34^^,^^35^ it is plausible that receptor crosstalk also exists in these cells. Whether GABA_B_R signaling is susceptible to rapid loss of cell-surface receptors in SST INs, and if this affects cell function and the local circuit, remains unknown.

We hypothesized that GABA_B_R activation regulates its own surface expression, favoring greater plasticity in SST INs. To determine this, we performed quantitative SDS-digested freeze-fracture replica immunogold labeling (SDS-FRL) electron microscopy, *ex vivo* electrophysiological recordings from CA1 of the mouse hippocampus following prolonged application of the selective GABA_B_R agonist baclofen, and behavioral characterization. We found, in opposition to our hypothesis, that after sustained GABA_B_R activation, synaptic plasticity of SST INs was reduced, independent of direct receptor activation. This reduction in plasticity was due to concomitant protein phosphatase 2A (PP2A)-dependent decrease in GABA_B_Rs, Ca_v_1.2 VGCCs, and mGluR1α—all of which are required for LTP of SST INs. Finally, we show that baclofen administration leads to circuit level strengthening of long-range inputs to CA1, which are dependent on SST IN plasticity.

## Results

In this study, we tested the hypothesis that sustained baclofen application-induced activation of GABA_B_Rs resulting in downregulation of surface expression of the receptor, and thus favoring enhanced plasticity of SST INs. To investigate this mechanism, we performed quantitative immunoelectron microscopy (iEM) and electrophysiology in adult mice.

### Native GABA_B_Rs, mGluR1α, and L-type calcium channels are downregulated in SST in membranes following baclofen pre-application

We have previously shown that mouse SST IN dendrites possess a high density of GABA_B1_, Ca_V_1.2, and mGluR1α.^11^ The latter two proteins are required for associative theta-burst stimulus (aTBS) LTP of SST INs,^36^^,^^37^ and is inhibited by GABA_B_Rs.^11^ To determine whether pre-application of the GABA_B_R agonist baclofen (20 μM) altered membrane localization of GABA_B_Rs as compared to mGluR1α and Ca_V_1.2, we performed SDS-FRL iEM analysis from acute mouse hippocampal slices pre-treated with baclofen (*n* = 5 mice), compared slices treated with vehicle (*n* = 5 mice) or the GABA_B_R antagonist CGP-55,845 (5 μM, *n* = 4 mice; Figure 1A). All SDS-FRL data are shown as animal average values, with statistics shown based on linear mixed-effects modeling to account for inter-animal variability. Data from each dendritic profile within animal is shown in Figure S1.

**Figure 1:**
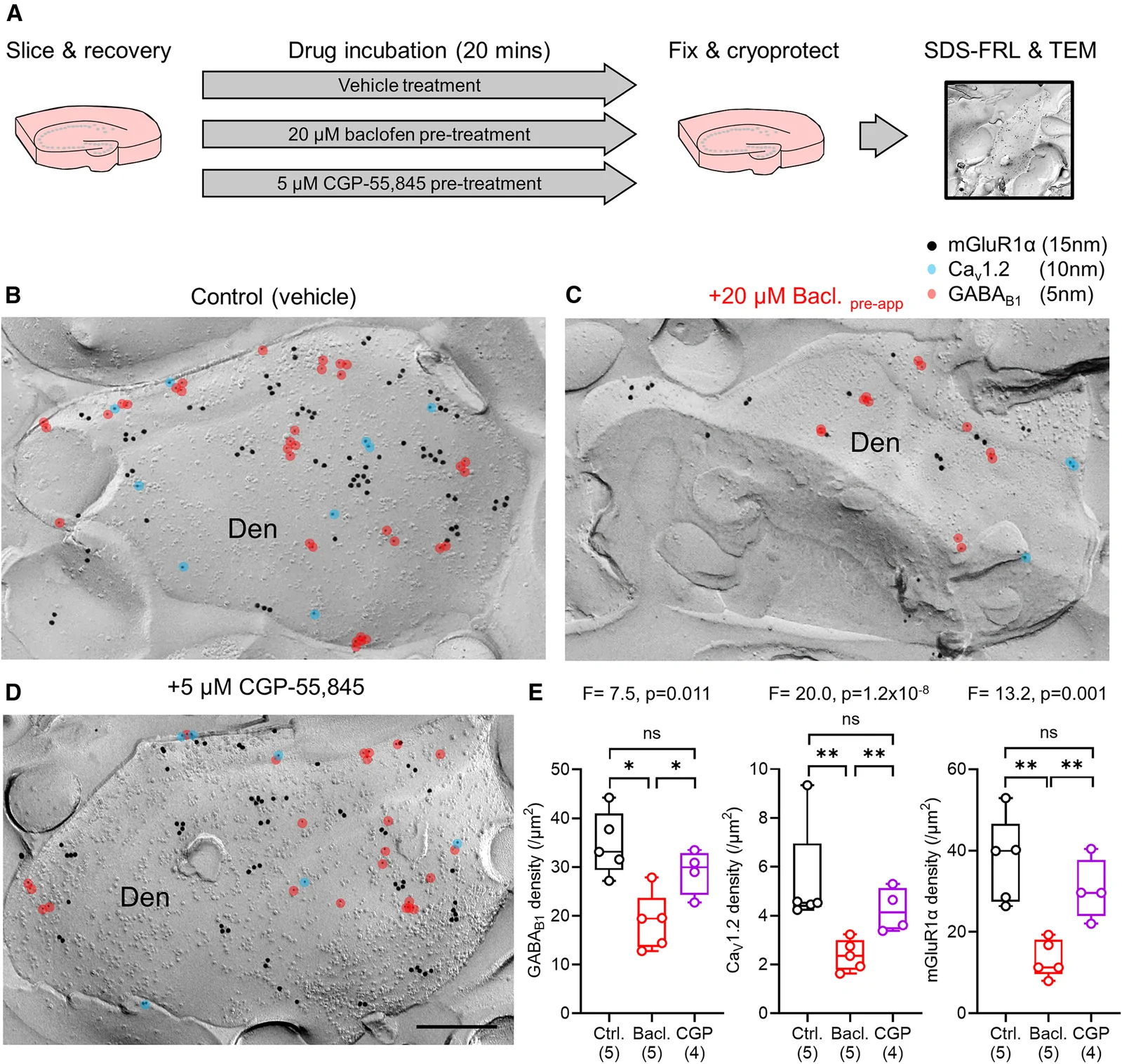
Sustained GABA_B_R activation leads to reduction of membrane-associated GABA_B1_, Ca_V_1.2, and mGluR1α proteins from putative SST INs (A) Schematic of the experimental overview, indicating treatment and experimental sample times. (B) Example electron micrographs of SDS-FRL for mGluR1α (15 nm immunoparticles), Ca_V_1.2 (10 nm, blue overlay) and GABA_B1_ (5 nm, red overlay) on a putative SST IN dendrite in *str. O/A* of CA1 in a vehicle slice. The same labeling but performed in brain slices following 20 min baclofen pre-application (20 μM), (C) or CGP-55,845 (5 μM), (D). (E) Quantification of labeling density for GABA_B1_ (left, control: 54 dendrites, 5 mice; baclofen: 78 dendrites, 5 mice; CGP: 74 dendrites, 4 mice), Ca_v_1.2 (middle, control: 68 dendrites, 5 mice; baclofen: 78 dendrites, 5 mice; CGP: 74 dendrites, 4 mice), and mGluR1α (right, control: 77 dendrites, 5 mice; baclofen: 78 dendrites, 5 mice; CGP: 74 dendrites, 4 mice), in slices treated with vehicle (Ctrl., black), baclofen pre-application (Bacl.), or CGP-55,845 (CGP). All data are shown as boxplots depicting the 25%–75% range with median, maximum range, and data from individual mice overlaid as open circles. Statistics shown as: ns, not significant; *p* > 0.05, ∗*p* < 0.05, ∗∗*p* < 0.01, ∗∗∗*p* < 0.001; all from post hoc Tukey’s tests following LMM analysis. Scale bars represent 200 nm for all images.

Under control conditions (Figure 1B), putative SST IN dendrites were identified based on high levels of surface labeling for mGluR1α,^34^ with an average density of 37.8 ± 4.8 particles/μm^2^ (Figures 1B and E). Consistent with our previous data in rats,^11^ mGluR1α-positive dendrites displayed high-density GABA_B1_ subunit labeling of 34.8 ± 2.9 particles/μm^2^ and moderate expression of the L-type channel subunit Ca_V_1.2 at 5.43 ± 0.98 particles/μm^2^ (Figures 1B and 1E). Compared to control slices, baclofen pre-application led to a 46% reduction in density to 18.8 ± 2.6 particles/μm^2^ for GABA_B1_ (t_(5,5)_ = 3.52, *p* = 0.014, Tukey’s test; Figures 1C and 1E). We observed a 56% reduction in Ca_V_1.2 to 2.38 ± 0.29 particles/μm^2^ (t_(5,5)_ = 5.77, *p* = 0.001, Tukey’s test) and a 65% reduction of mGluR1α labeling 13.3 ± 2.1 particles/μm^2^ (t_(5,5)_ = 4.82, *p* = 0.002, Tukey’s test) (Figures 1C and 1E). Application of the GABA_B_R antagonist CGP-55,845 did not decrease density of GABA_B1_ (t_(5,4)_ = 0.69, *p* = 0.77, Tukey’s test), Ca_V_1.2 (t_(5,4)_ = 1.69, *p* = 0.27, Tukey’s test) or mGluR1α (t_(5,4)_ = 0.84, *p* = 0.69, Tukey’s test) relative to control levels (Figures 1D and 1E). This effect on effector channels appeared confined to SST INs, as measurement of CA1 PC dendrites in the same replicas revealed that while baclofen pre-application reduced GABA_B1_ density by 49.9% (F_(5,5)_ = 45.22, *p* = 4.57 × 10^−10^, linear-mixed effects modeling (LMM, Figure S2) Ca_V_1.2 was unchanged (F_(3,3)_ = 0.009, *p* = 0.93, LMM; Figure S2). mGluR1α was not tested as this protein is not expressed abundantly on CA1 PCs. Together, these data reveal that GABA_B_R activation leads to its own reduction from the membrane of SST INs, but also leads to downregulation of Ca_V_1.2 and mGluR1α density.

### Baclofen pre-application leads to reduced Ca_v_1.2 and group 1 mGluR-mediated currents in SST INs

To determine whether baclofen pre-application regulates the function of Ca_v_1.2 and mGluR1α in SST INs, we next performed whole-cell patch clamp recordings from identified cells using targeted pharmacology to interrogate receptor and channel loss (Figure 2A), and determine whether this is dependent on PP2A.^26^^,^^31^^,^^38^

**Figure 2:**
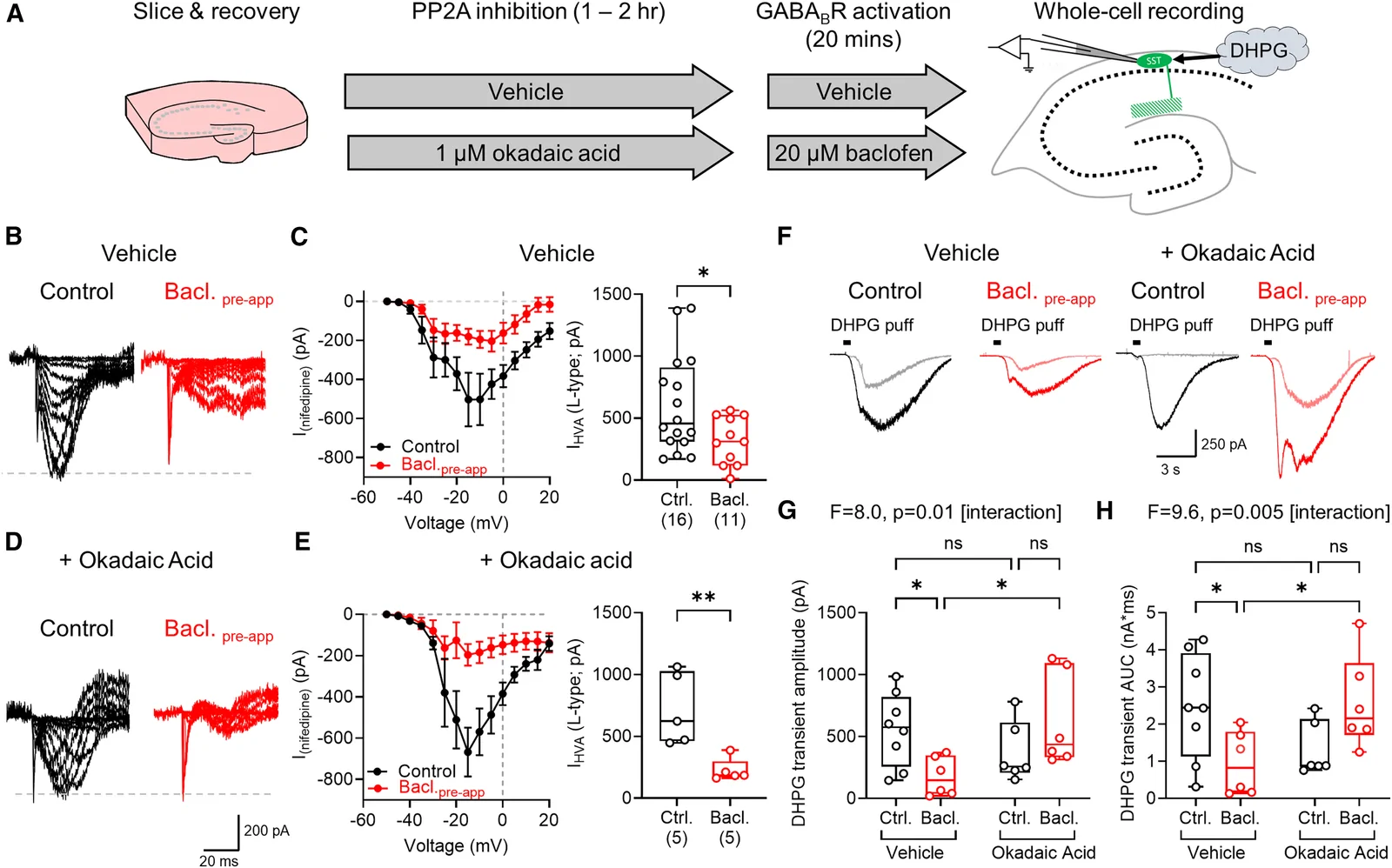
Ca_V_1.2 and group 1 mGluR-mediated currents are reduced following baclofen pre-application (A) Schematic of recording conditions, highlighting treatment periods and recording times. (B) Example nifedipine-sensitive VGCC current responses recorded in SST INs following depolarizing stimulation from −60 mV. Dashed line indicates control response amplitude for comparison. (C) Quantification of voltage-dependency of nifedipine-sensitive VGCC currents (left) and peak nifedipine-sensitive currents (right) from control (black, 16 cells) and baclofen pre-application (11 cells). (D) Example nifedipine-subtracted isolated VGCC current responses in SST INs pre-application with 1 μM okadaic acid. (E) Quantification of voltage-dependency of nifedipine-sensitive VGCC currents (left) and peak nifedipine-sensitive currents (right) in OA pre-application SST INs; from control (black, 5 cells) and baclofen pre-application (5 cells). (F) Example voltage responses following 50 μM S-DHPG puff application to SST INs, recorded under control conditions (black) or following baclofen pre-application (red), following either vehicle or 1 μM OA pre-application. (G) Quantification of absolute current amplitudes following S-DHPG puff. Data from vehicle: control (black, 8 cells) and baclofen pre-application (6 cells); and OA-treated: control (black, 6 cells) and baclofen pre-application (6 cells). (H) Quantification of the area-under-curve (AUC) for S-DHPG puff induced transients. Data from vehicle: control (black, 8 cells) and baclofen pre-application (6 cells); and OA-treated: control (black, 6 cells) and baclofen pre-application (6 cells). All data are shown as either mean ± SEM (current-voltage plots) or boxplots showing 25%–75% box with median, with maximum range shown. Individual cell data are shown overlaid. Statistics shown as: ns, not significant; *p* > 0.05, ∗*p* < 0.05; from two-way ANOVA and Mann-Whitney non-parametric tests.

As Ca_v_1.2 containing VGCCs are a major target of GABA_B_R-mediated inhibition in SST INs,^11^ we first performed whole-cell recordings using a Cs-gluconate-based internal solution containing QX-314 (5 mM) to block voltage activated K^+^ and Na^+^ channels, respectively, in the presence of CNQX (10 μM), DL-AP5 (50 μM), and gabazine (10 μM) to block ionotropic receptor signaling. High-voltage-activated Ca^2+^ responses were measured from −60 mV voltage-clamp, in which 5 mV depolarizing current steps were applied, followed by bath application of the selective L-type VGCC blocker nifedipine (10 μM; Figure 2B). The average subtracted L-type current measured across a range of depolarizing stimuli revealed large amplitude currents in SST INs recorded under control conditions, which were substantially reduced, but with a similar voltage-dependency in slices pre-application with baclofen. We found that baclofen pre-application reduced the peak L-type current measured by 37% (U_(16,11)_ = 50; *p* = 0.032, Mann-Whitney test, Figure 2C). We performed the same recordings, but in slices that had been incubated with the PP2A inhibitor okadaic acid (OA, 1 μM; Figure 2D). Blocking PP2A activity had minimal effect on current-voltage response or peak L-type currents following baclofen pre-application (U_(5,5)_ = 0; *p* = 0.008, Mann-Whitney; Figure 2E).

For mGluR1 signaling, we recorded SST INs in whole-cell configuration then puff applied the selective, high-affinity group I mGluR agonist S-dihydroxyphenylglycine (DHPG; 50 μM, 200 ms, and 10 PSI) within 100–200 μm of the cell body. S-DHPG puffs resulted in large and slow depolarizing currents in SST INs, which were partially sensitive to the mGluR1α selective antagonist LY367,385 (100 μM). Measurement of the same S-DHPG-induced currents in slices that were pre-application with baclofen possessed typically smaller currents (Figure 2F). Quantification of peak S-DHPG currents revealed that baclofen pre-application reduced the amplitude (F(1,22) = 7.99, *p* = 0.01, two-way ANOVA [interaction], Figure 2G) and the charge transfer (F(1,22) = 9.59, *p* = 0.005, two-way ANOVA [interaction], Figure 2H) effects, which were not present when slices were incubated with OA prior to baclofen pre-application.

### Baclofen pre-application-dependent reduction in Ca_v_1.2 and mGluR1α surface density are mediated by PP2A

To confirm surface downregulation of GABA_B_Rs was indeed dependent on PP2A in SST INs, we used a mouse line expressing a channelrhodopsin-2 fused to yellow fluorescence protein (ChR2/YFP) under the SST promotor, enabling us to selectively identify SST IN dendrites^10^ in SDS-FRL iEM. Initially, we prepared slices from WT mice treated with the PP2A inhibitor OA. However, in these slices we observed minimal to no labeling for mGluR1α, GABA_B1_ or Ca_V_1.2 in putative SST IN and CA1 PC dendritic profiles (Figure S3). This effect was observed across >5 independent experiments.

As such, we next used the small-molecule PP2A inhibitor fostriecin (50 nM), with which slices were incubated with for 1 h prior to baclofen pre-application (Figure 3A). In *str. O/A* from the ChR2/YFP mice, we observed strong immunolabeling for YFP (33.7 ± 1.07 particles/μm^2^, Figure 3B). In vehicle slices, we observed a 51% reduction of GABA_B1_ surface density following baclofen pre-treatment (17.7 ± 4.5 particles/μm^2^, t_(3,3)_ = 3.9, *p* = 0.012, Kenward-Roger test, Figures 3B and 3D), consistent with observations from mGluR1α-labeled dendrites (Figure 1). Incubation with fostriecin prevented the baclofen pre-treatment-mediated reduction in GABA_B1_ surface density (32.0 ± 1.7 particles/μm^2^, t_(3,3)_ = 0.47, *p* = 0.66, Kenward-Roger test, Figures 3C and 3D), confirming that PP2A inhibition prevents GABA_B_R surface downregulation.

**Figure 3:**
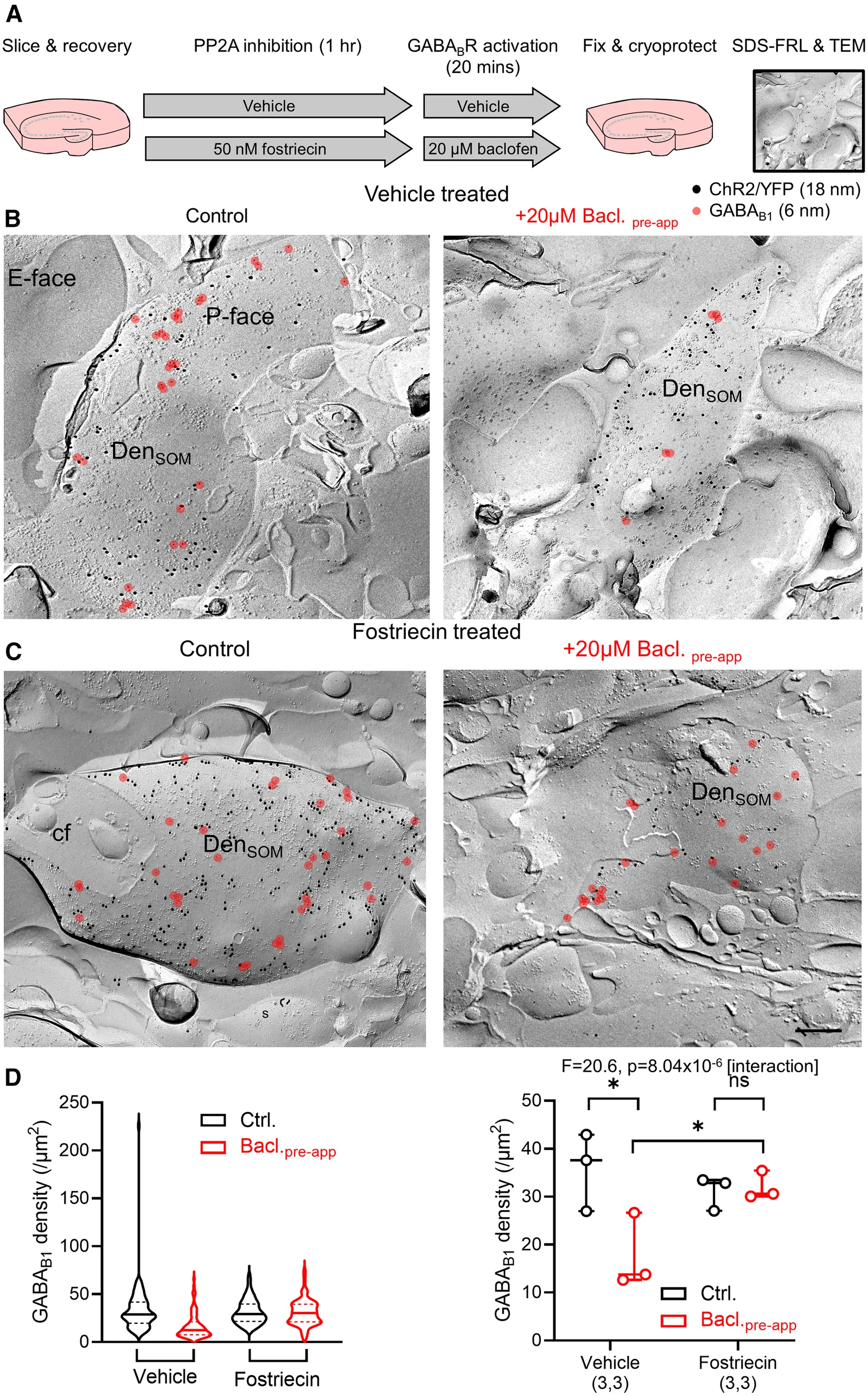
Inhibition of PP2A prevents reduction of surface GABABR expression in SST INs following their activation (A) Schematic of experimental conditions, highlighting treatment periods and fixation times. (B) Example electron micrographs of SDS-FRL for SST-cre dependent expression of ChR2/YFP (18 nm immunoparticles) and GABA_B1_ (6 nm, red overlay) in *str. O/A* of CA1 of slices with vehicle or baclofen pre-application (20 μM). (C) The same labeling but performed in slices incubated with 50 nM fostriecin for 1 h prior to baclofen pre-application. (D) Quantification of GABA_B1_ density in vehicle slices (Ctrl., black, 79 dendrites [vehicle], 70 dendrites [fostriecin], both 3 mice) or those pre-application with baclofen (Bacl., red, 87 dendrites [vehicle], 81 dendrites [fostriecin], both 3 mice) with and without fostriecin incubation. All data are shown as violin-plots and boxplots depicting the 25%–75% range with median, maximum range shown, and average density calculated from individual dendrites or mice results overlaid as open circles. Scale bars represent 200 nm.

To determine whether GABA_B_R-dependent loss of L-type VGCCs also requires PP2A-dependent mechanisms, we performed the same experiments but labeling for Ca_V_1.2 (Figure 4A). In replicas from vehicle-treated slices, we observed Ca_V_1.2 labeling of 3.75 ± 0.40 particles/μm^2^, which was 45% reduced in baclofen pre-application slices (2.07 ± 0.24 particles/μm^2^, t_(3,3)_ = 3.51, *p* = 0.010, Kenward-Roger test, Figures 4B and 4D), consistent with mGluR1α immunolabeling experiments (Figure 1). Fostriecin pre-incubation (Figure 4C) prevented this reduction in Ca_V_1.2 expression following baclofen treatment (3.74 ± 0.24 particles/μm^2^, t_(3,3)_ = 0.88, *p* = 0.41, Kenward-Roger test, Figures 4C and 4D), confirming that PP2A inhibition prevents removal of plasma membrane-localized Ca_v_1.2 channels.

**Figure 4:**
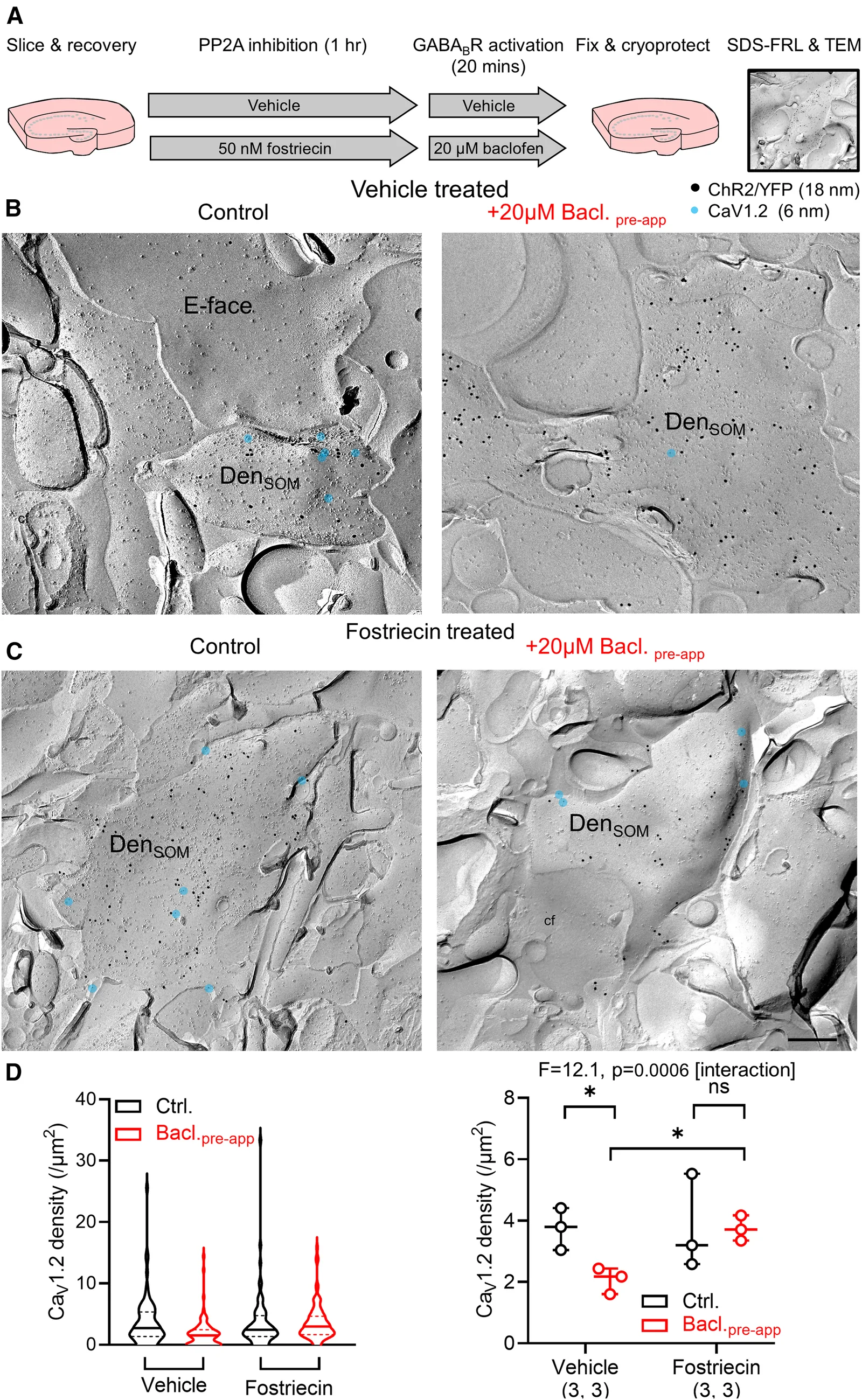
Inhibition of PP2A prevents downregulation of CaV1.2 from SST IN membranes following GABABR activation (A) Schematic of experimental conditions, highlighting treatment periods and fixation times. (B) Example electron micrographs of SDS-FRL for SST-cre dependent expression of ChR2/YFP (18 nm immunoparticles) and Ca_V_1.2 (6 nm, blue overlay) in *str. O/A* of CA1 of slices with vehicle or baclofen (20 μM). (C) The same labeling but performed in slices pre-application with 50 nM fostriecin for 1 h prior to baclofen application. (D) Quantification of Ca_V_1.2 density in slices treated with vehicle (Ctrl., black, 74 dendrites [vehicle], 69 dendrites [fostriecin], both 3 mice) or baclofen (Bacl., red, 86 dendrites [vehicle], 86 dendrites [fostriecin], both 3 mice) with and without fostriecin pre-application. All data are shown as violin-plots and boxplots depicting the 25%–75% range with median, maximum range shown, and average density calculated from individual dendrites or mice results overlaid as open circles. Scale bars represent 200 nm.

These data confirm that baclofen-induced reduction of GABA_B_R surface expression requires PP2A in SST INs in *str. O/*A, and that inhibiting this prevents loss of Ca_V_1.2 from their dendritic membranes.

### Sustained GABA_B_R activation leads to reduced plasticity of SST INs that is mediated by receptor loss

We have previously shown that aTBS-induced LTP in SST INs can be prevented by acutely activating GABA_B_Rs.^11^ However, our data suggest that application of baclofen leads to reduced LTP machinery from SST INs. To determine whether sustained activation of GABA_B_Rs shape the long-term plasticity landscape of SST INs, we first performed whole-cell patch-clamp recordings in *str. O/A* from adult male and female transgenic mice expressing YFP (Venus variant) under the vesicular GABA transporter (vGAT) promoter (Figure 5A). We recorded 108 *str. O/A* INs for the current study, those that were recovered (*n* = 70) typically had horizontal dendrites confined to *str.* *oriens* (*str.* *ori*), with axons projecting to *str. L-M* and were uniformly SST immunoreactive (Figure 5B). Recorded SST INs displayed prominent I_h_-mediated sag potentials, and possessed a medium to high rate of action potential (AP) discharge, which was lower after baclofen pre-application (Figure 5C) due to reduced input resistance and elevated rheobase (Table S1).

**Figure 5:**
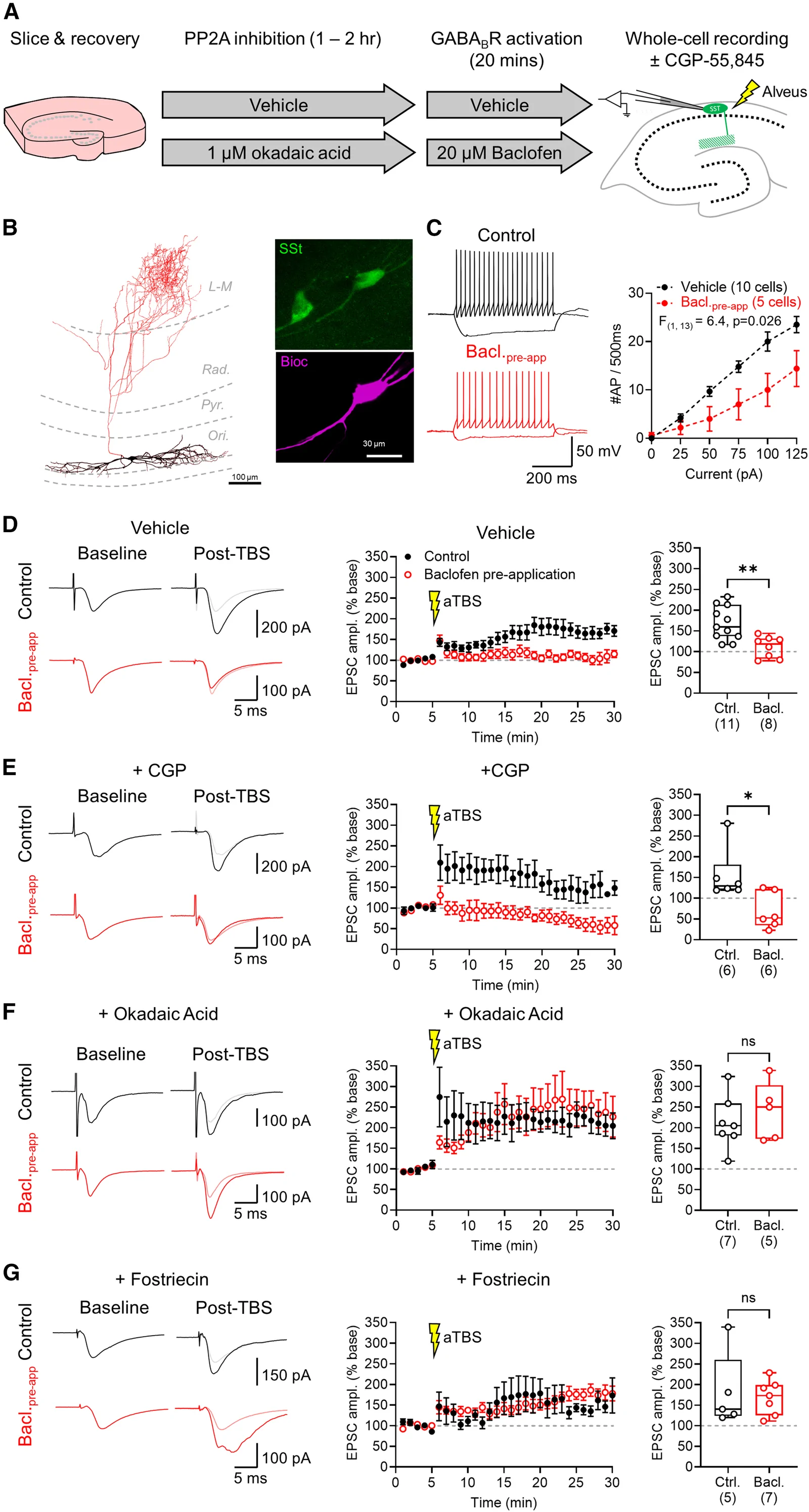
LTP of local inputs to SST INs is abolished by prolonged baclofen application (A) Schematic of recording conditions, highlighting treatment periods and recording times. (B) Example reconstruction of an SST IN recorded from *str. O/A* of the adult mouse CA1 region, showing somatodendritic (black) and axonal (red) ramifications. Inset immunoreactivity for SST (green) and biocytin (purple). Scale bar shown as 100 μm. (C) Voltage responses in response hyper-to depolarizing current steps (−500 to +500 pA, 100 pA steps, 500 ms duration) from SST INs recorded in following vehicle (black, 10 cells) and baclofen-pre-application (Bacl._pre-app_; red, 5 cells). Right, quantification of the action potential output of SST INs recorded under both conditions. (D) Example excitatory postsynaptic current (EPSC) traces from vehicle (upper, black) and 20 μM baclofen pre-application (lower, red) measured under control conditions; for aTBS-LTP traces baseline EPSCs are shown underlain (gray and pink). Middle, time-course of plot of EPSC amplitude before and after aTBS-LTP induction for vehicle (black) and baclofen pre-application (red); 100% baseline level is shown for reference (gray dashed line). Right, bar-chart of EPSC amplitude change post aTBS-LTP (25–30 min) compared to baseline for vehicle (black, 11 cells) and baclofen pre-application (red, 8 cells); 100% baseline level indicated (gray dashed line). (E) aTBS-LTP recordings from SST INs performed in the presence of 5 μM CGP-55,845 (CGP) according to the same scheme as (D), but from 6 cells (control) and 6 cells (baclofen). (F) aTBS-LTP following pre-application of slices with 1 μM okadaic acid for 1 h with subsequent vehicle or baclofen pre-application, from 7 cells (control) and 5 cells (baclofen). (G) aTBS-LTP in slices pre-application with 50 nM fostriecin for 1 h prior to vehicle or baclofen pre-application, from 5 cells (control) and 7 cells (baclofen). All data are shown as either mean ± SEM (time-course plots) or boxplots showing 25%–75% box with median, with maximum range shown and individual cell results. Statistics shown as: ns, not significant; *p* > 0.05, ∗*p* < 0.05, ∗∗*p* < 0.01 from Mann-Whitney non-parametric tests.

Under vehicle control conditions, we observed excitatory postsynaptic currents (EPSCs) resulting from *alveus* stimulation of 197.8 ± 17.6 pA (13.3 ± 3.9 V stimulation, *n* = 11 SST INs), which following aTBS-LTP induction led to a facilitation of 68.5% ± 12.9% above baseline levels (*p* = 0.001, Wilcoxon matched-pairs test). In baclofen pre-application slices, we observed baseline EPSCs with an average amplitude of 195.6 ± 54.6 pA (8.5 ± 2.9 V stimulation, *n* = 8 SST INs), which did not differ from vehicle recordings in amplitude (U_(11, 8)_ = 28, *p* = 0.21, Mann-Whitney) or stimulation strength (U_(11, 8)_ = 33, *p* = 0.38, Mann-Whitney). An aTBS-LTP induction in baclofen pre-application slices failed to increase EPSC amplitude above baseline levels (11.3% ± 9.6% above baseline, *p* = 0.38, Wilcoxon matched-pairs test), and which was lower than for vehicle conditions (U_(11,8)_ = 10; *p* = 0.004, Mann-Whitney). To confirm that this loss of aTBS-LTP was not due to direct activation of GABA_B_Rs, we performed the same experiments, but where the GABA_B_R antagonist CGP-55,845 (CGP, 5 μM) was applied throughout baseline recording and LTP induction and maintenance (Figure 5E). In the presence of CGP, we still failed to induce aTBS-LTP in baclofen pre-application slices (31.3% ± 19.6% below baseline, *p* = 0.31, Wilcoxon matched-pairs test), despite persistent induction in vehicle-treated slices (54.5% ± 28.0% above baseline *p* = 0.031, Wilcoxon matched-pairs test; U_(6,6)_ = 5; *p* = 0.041 Mann-Whitney), confirming that aTBS-LTP inhibition was not due to direct receptor activation. Interestingly, we found that in SST IN LTP induced in the presence of CGP did not differ from control (U_(11,6)_ = 23; *p* = 0.35, Mann-Whitney), indicating that GABA_B_Rs minimally control LTP induction under baseline conditions.

Postsynaptic GABA_B_Rs undergo PP2A-dependent control of internalization.^26^^,^^30^^,^^31^^,^^38^ We next asked if impaired aTBS-LTP in SST INs was due to PP2A-dependent mechanisms. For these experiments, we incubated slices with OA (1 μM) for 60–120 min prior to vehicle or baclofen pre-application. Following OA incubation, we continued to observe reliable aTBS-LTP in both vehicle slices (112.1% ± 26.4% above baseline, *p* = 0.016 Wilcoxon paired test) and baclofen pre-application slices in SST INs (140.1% ± 31.3% above baseline, *p* = 0.029 Wilcoxon paired test), which were not different (U_(7,5)_ = 15; *p* = 0.76, Mann-Whitney test; Figure 5F). As our SDS-FRL labeling was not possible with OA ( Figure 3), we next confirmed that fostriecin (50 nM) produced similar effects to OA. After fostriecin incubation (60–90 min), we observed a tendency toward aTBS-LTP in vehicle slices (81.8% ± 45.6% above baseline, *p* = 0.063 Wilcoxon paired test) and robust LTP in baclofen pre-application slices (69.0% ± 17.0% of baseline, *p* = 0.016 Wilcoxon paired test), which was not different from vehicle (U_(5,7)_ = 16, *p* = 0.876, Mann-Whitney test; Figure 5G).

Combined, these data indicate that sustained GABA_B_R activation impairs aTBS-LTP induction at CA1 PC inputs onto SST INs, which is likely independent of receptor activation during LTP induction and which likely relies on PP2A-dependent downregulation of membrane anchored Ca_V_1.2 and mGluR1α.

### Prolonged activation of GABA_B_Rs impairs presynaptic release of GABA, but not postsynaptic expression of GABA_B_Rs in CA1 PCs

Assessing the wider function of GABA_B_R signaling following sustained pharmacological activation is critical to infer circuit wide effects. In CA1 PCs, following baclofen pre-application, we observed a tendency toward reduced GABA_B1_, but unchanged Ca_V_1.2 surface expression. We next determined if these effects altered inhibitory signaling in and onto CA1 PCs. Therefore, we recorded GABA_B_R-mediated slow inhibitory postsynaptic currents (IPSCs) in the presence of CNQX (10 μM), DL-AP5 (50 μM), and gabazine (10 μM) to block ionotropic receptor signaling (Figure 6A). We found that slow IPSCs were substantially reduced following baclofen pre-application (U_(10,7)_ = 3, *p* = 0.0007, Mann-Whitney test; Figure 6B). However, baclofen-mediated whole-cell currents (I_WC_) were unaffected by baclofen pre-application (Figure 6C), with normal peak current-density (U_(9,7)_ = 15.5, *p* = 0.10, Mann-Whitney test; Figure 6D). One explanation for this disconnect between synaptic and pharmacological activation of GABA_B_Rs could be impaired GABA release. As such, we measured the input/output (I/O) relationship of ionotropic GABA_A_R-mediated IPSCs following *str. L-M* stimulation as a proxy for synaptic strength. We found that for the same stimulus, fast IPSCs were consistently lower in amplitude, which upon quantification revealed 46% lower I/O slopes (U_(7,6)_ = 4, *p* = 0.014, Mann-Whitney test, Figure 6E). This loss of GABA strength was likely due to impaired presynaptic release, as paired-pulse ratio (PPR) was significantly elevated in the baclofen pre-application group (U_(7,6)_ = 1, *p* = 0.0023, Mann-Whitney test, Figure 6F). To determine whether baclofen pre-application alters presynaptic GABA_B_R function, we next bath applied 10 μM baclofen to recordings of fast IPSCs (Figure 6G). Slices with pre-application of baclofen tended to have lower GABA_B_R presynaptic inhibition of IPSCs in *str. L-M* (U_(7,6)_ = 7, *p* = 0.051, Mann-Whitney test, Figure 6H). Finally, we compared the effect of baclofen pre-application on CA1 PC intrinsic excitability. Contrary to what we observed in SST INs, we find that CA1 PCs are largely unaffected by baclofen pre-application, with subtle effects on membrane capacitance and AP kinetics observed (Table S2).

**Figure 6:**
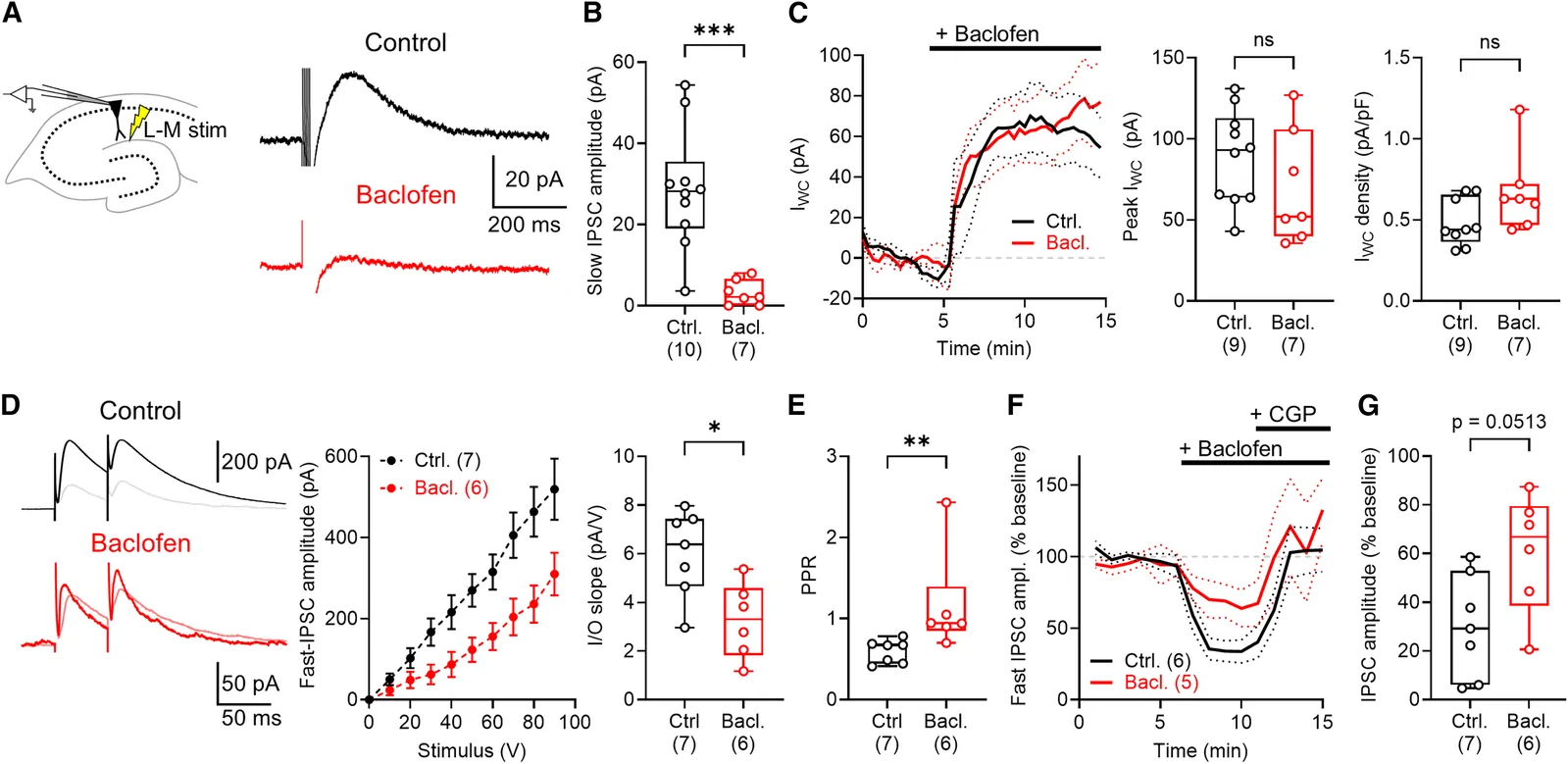
Prolonged GABA_B_R activation decreases GABA release in str. L-M, specific for presynaptic but not postsynaptic GABA_B_R-mediated inhibition (A) Experimental schematic of recordings from CA1 PCs and example slow IPSCs recorded following 5 × 200 Hz stimulation of *str. L-M* in the presence of CNQX (10 μM), DL-AP5 (50 μM) and gabazine (10 μM) from −65 mV voltage-clamp from control slices (black) and those pre-application with 20 μM baclofen (red). (B) Quantification of slow inhibitory postsynaptic current (IPSC) amplitudes from control and baclofen pre-application slices, from 10 cells (control) and 7 cells (baclofen pre-application). (C) Time course of whole-cell current (I_WC_) recorded from −65 mV following bath application of 10 μM baclofen in control and baclofen pre-application slices. Quantification of I_WC_ current density, normalized to cell capacitance from 9 cells (control) and 7 cells (baclofen pre-application). (D) Fast IPSCs evoked following paired stimulation of *str. L-M* from 0 mV using Cs-gluconate internal solution in the presence of CNQX (10 μM), DL-AP5 (50 μM) from control and baclofen pre-application slices. Traces recorded following bath application of 10 μM baclofen are shown underlain (gray/pink). Right, input-output (I/O) plot of fast IPSC amplitude from both groups. Right, quantification of I/O slope for fast IPSCs. Data from 7 cells (control) and 6 cells (baclofen pre-application). (E) Comparison of paired-pulse ratio (PPR) between treatment groups. Data from 7 cells (control) and 6 cells (baclofen pre-application). (F) Time course of fast IPSC amplitude before, and following bath application of 10 μM baclofen and 5 μM CGP-55,845 (CGP) in control and baclofen pre-application slices. (G) Quantification of baclofen mediated inhibition of fast IPSC amplitudes, expressed as % of baseline. Data from 7 cells (control) and 6 cells (baclofen pre-application). Data are shown as either mean ± SEM (C, E, and G) or boxplots showing 25%–75% box with median, with maximum range (B, D, F, and H). Individual cell data are shown overlaid. Statistics shown as: ns, not significant; *p* > 0.05, ∗*p* < 0.05, ∗∗∗*p* < 0.001; from Mann-Whitney non-parametric tests.

Together these data suggest that prolonged GABA_B_R activation has limited effects on the postsynaptic function of CA1 PCs; in particular, we find no evidence for reduced functional GABA_B_R expression. However, due to strong reductions in presynaptic GABA release, postsynaptic IPSCs are functionally reduced.

### GABA_B_R-dependent loss of LTP proteins in SST INs leads to enhanced temporoammonic plasticity onto CA1 PCs

SST INs are known to control the ability of CA1 PCs to undergo plasticity, notably in their distal dendrites in *str. L-M* aligned with entorhinal cortex inputs.^23^ To determine whether prolonged activation of GABA_B_Rs, and subsequent loss of SST IN plasticity alter temporoammonic LTP, we performed extracellular field recordings from *str. L-M* of CA1, in which we induced plasticity at SST INs via repetitive stimulation of the alveus, followed by high-frequency stimulation (HFS, 1 × 100 Hz) to the temporoammonic pathway in vehicle or those with pre-application of 20 μM baclofen for 20 min (Figure 7A). Following HFS to *str. L-M*, we observed increased field excitatory postsynaptic potential (fEPSP) amplitudes when measured in all recordings from both treatment groups (Figure 7B), which when measured at 50–60 min post induction were 25.6% ± 7.8% above baseline in vehicle control recordings, and which did not differ in baclofen pre-application slices (20.5% ± 10.4%; U_(15,19)_ = 104, *p* = 0.19, Mann-Whitney test, Figure 7C). However, we noted that a higher proportion of slices pre-application with baclofen failed to undergo LTP (>10% above baseline) as compared to vehicle controls (χ^2^ = 7.5, *p* = 0.0062 chi-squared test; Figure 7D). Therefore, we next analyzed only those recordings in which LTP was observed (Figure 7E). Further quantification of successful LTP recordings (Figure 7F) revealed that post-tetanic potentiation (0–1 min post HFS) was 34% higher in baclofen pre-application slices (U_(11,10)_ = 25, *p* = 0.018, Mann-Whitney test) and fEPSPs measured at 20–30 min (consistent with whole-cell recordings) were also 24% higher than control slices (U_(12,10)_ = 27, *p* = 0.015, Mann-Whitney test), while LTP measured at 50–60 min was higher, albeit not significantly so (U_(12,10)_ = 37, *p* = 0.07, Mann-Whitney test). We observed no difference in fEPSP slope relative to stimulus strength between control and baclofen pre-application slices (Figure 7G). These effects were likely specific to SST INs, as performing the same recordings but in the presence of the mGluR1α-specific antagonist LY367,385 (100 μM), prevented any baclofen pre-application effects on temporoammonic LTP (Figure S4). This also excludes the effects of baclofen pre-application on GABAergic release and CA1 PC excitability as being causal to this effect. Furthermore, contextual fear conditioning, a learning paradigm that requires SST IN plasticity was impaired when baclofen was administered 1 h prior to conditioning (Figure S5).

**Figure 7:**
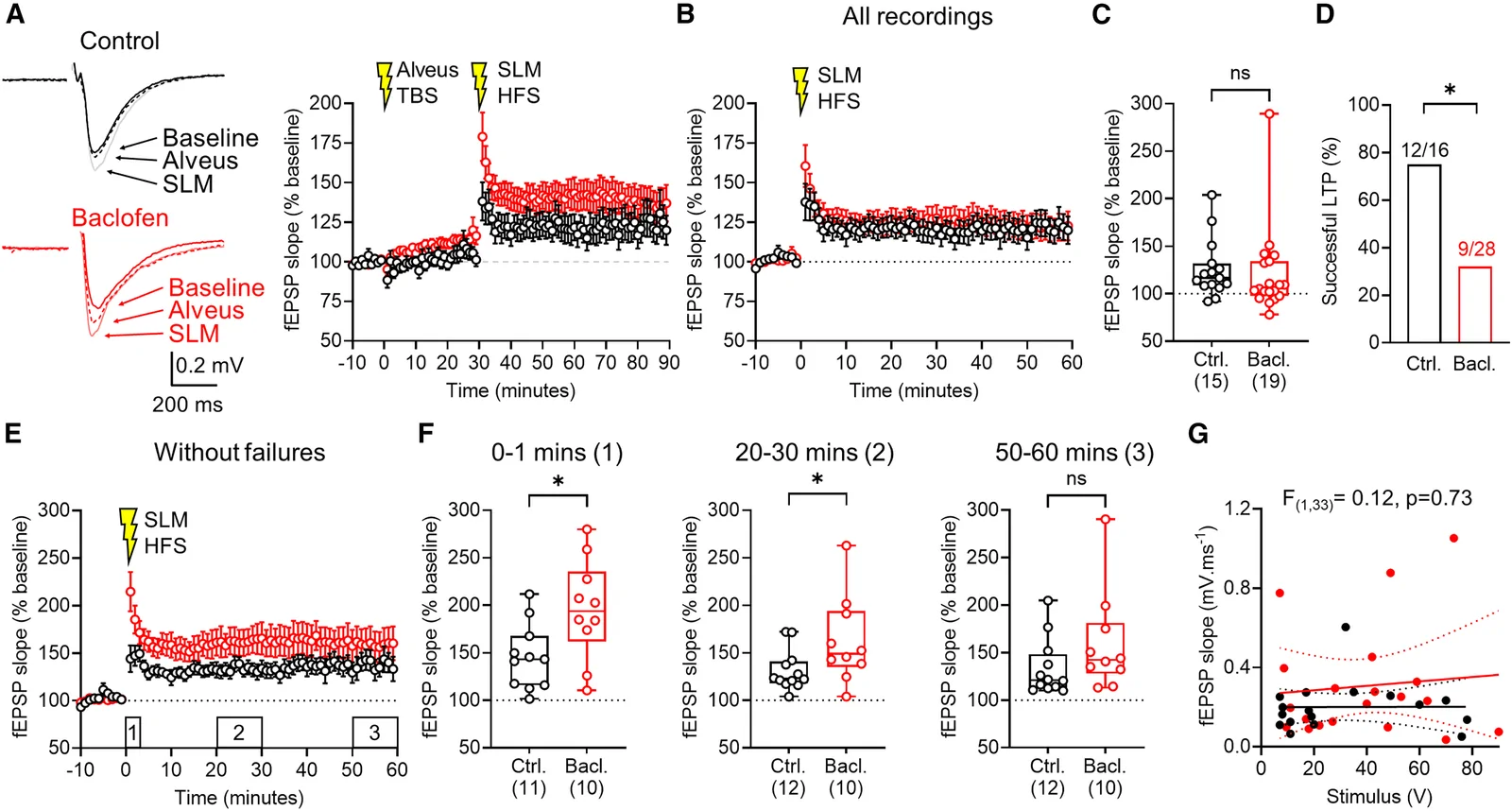
Prior GABA_B_R activation abolishes SST IN inhibition of short-term temporoammonic (TA) LTP (A) Example field excitatory postsynaptic potential (fEPSP) responses recorded in *str. L-M* following temporoammonic stimulation, from control (black) and 20 μM baclofen pre-application (red) slices. Traces are shown for initial baseline (solid line), following *alveus* LTP induction (dashed lines) and following *str. L-M* 1 × 100 Hz HFS (gray/pink lines). Right, summary time-course of all fEPSP recordings normalized to the initial baseline, indicating LTP induction points (lightning bolts). (B) Time-course of LTP induced in *str. L-M* (SLM, lightning bolt), normalized to the 20–30 min baseline following *alveus* stimulation in all recordings. (C) Magnitude of fEPSP facilitation at 50–60 min post str. *L-M* HFS in all recordings. Data from 15 slices (control) and 19 slices (baclofen pre-application). (D) Proportion of recordings in both control and baclofen pre-application that successfully induced LTP (>10% facilitation at 50–60 min post HFS). (E) Time-course of LTP induced in *str. L-M* normalized to the 20–30 min baseline following *alveus* stimulation in recordings where successful LTP was induced. (F) Quantification of fEPSP facilitation in control and baclofen pre-application slices at 0–1 (left), 20–30 (middle), and 50–60 (right) minutes post *str. L-M* HFS. Data from 12 slices (control) and 10 slices (baclofen pre-application. (G) Comparison of fEPSP slope and stimulus strength for all recordings in both groups. Data are shown as either mean ± SEM (A, B, E) or boxplots showing 25%–75% box with median, with maximum range (C and F), proportions (D), or as individual data (G). Individual cell data are shown overlaid. Statistics shown as: ns, not significant; *p* > 0.05, ∗*p* < 0.05; from Mann-Whitney non-parametric tests.

These data confirm that prolonged GABA_B_R activation leading to a downregulation in surface expression of GABA_B1_, mGluR1α, and Ca_V_1.2 in SST INs has profound effects on the ability of the CA1 microcircuit to encode inputs in *str. L-M*.

## Discussion

In this study, we tested the hypothesis that sustained GABA_B_R activation leads to receptor loss in hippocampal SST INs, releasing inhibition of synaptic plasticity. We failed to support this hypothesis and show baclofen administration leads to reduced surface expression of GABA_B_Rs, Ca_V_1.2, and also mGluR1α in SST INs, which is prevented by inhibition of PP2A. The loss of large proportion of surface receptors and channels impairs the long-term ability of SST INs to undergo synaptic plasticity, which in turn shifts the balance of plasticity at temporoammonic inputs. These data reveal a novel mechanism of long-term disinhibition that has the potential to shape the plasticity landscape of the CA1 region over behaviorally relevant timescales.

### PP2A-dependent reduction of membrane localized GABA_B_R regulates SST in activity

SST INs are critical elements of cortical circuits, gating the strength of inputs, and their plasticity, arriving from entorhinal cortex,^19^ and associative inputs within the hippocampus itself.^22^^,^^24^ These effects are mediated by a complex interplay of direct inhibitory actions on postsynaptic CA1 PCs, via presynaptic inhibition of glutamatergic and GABAergic inputs and through regulation of astrocyte function.^39^ SST IN activity leads to profound disinhibition of the hippocampal circuit to strengthen CA3 inputs^22^ and limit CA1 PC dendritic function.^24^ Thus, plasticity of SST INs themselves may act to shift the balance of CA1 function,^23^ which has a direct effect on CA3-dependent behaviors involving contextual information.^40^ Our previous work showed that GABA_B_Rs acutely inhibit L-type VGCCs comprising Ca_V_1.2 subunits on SST INs to inhibit LTP,^11^ which led to our hypothesis that GABA_B_R downregulation would promote LTP induction, as this key inhibitory mechanism was lost. We have failed to prove this hypothesis, with the opposite appearing to be the case, due to coincident loss of the key LTP machinery, namely mGluR1α and L-type VGCCs; and which depends on activation of PP2A and that has been implicated in GABA_B_R phosphorylation in other brain areas.^26^^,^^30^^,^^31^^,^^38^ While we do not explicitly provide direct evidence for internalization of GABA_B_Rs, previous studies have confirmed that PP2A-dependent surface loss is mediated by internalization.^31^ A plausible alternative however may be that receptor expression is masked by oligomerization of GABA_B_R subunits, which may reduce antibody binding.^41^ This does not explain the concomitant-decreased surface density of Ca_V_1.2 and mGluR1α. Other fates of lost GABA_B_R, such as protein degradation or recycling would both require initial internalization to be implicated via clathrin-mediated endocytosis.^42^

How PP2A regulates GABA_B_R, Ca_V_1.2, and mGluR1α surface expression remains undetermined. Following GABA_B_R activation, adenylyl cyclase activity is inhibited, leading to lower cAMP levels and reduced protein kinase A (PKA) activation, which in turn limits the phosphorylation of Serine 892 on the GABA_B2_ subunit, destabilizing surface receptors.^43^ Additionally, PP2A dephosphorylates Serine 783 on the GABA_B1_ subunit, also leads to internalization.^26^ As mGluR1α is a closely related type C G protein-coupled receptor (GPCR), it is plausible these receptors could be targeted through overlapping intracellular cascades,^44^ especially given PP2A is known to regulate recycling of mGluR1.^45^ For Ca_V_1.2, the situation is more complex, as inhibiting PP2A prevented its removal from the membrane of SST INs upon GABA_B_R activation, but nifedipine-sensitive currents were not restored. Given the close proximity of GABA_B1_ to Ca_V_1.2 in SST IN dendrites,^11^ it may bind to the GABA_B_R in SST INs and be co-internalized, as occurs for Kir3 channels in other brain regions.^46^ However, Ca_V_1.2 activity is upregulated by PKA phosphorylation and PP2A,^47^ thus inhibiting the latter may further dysregulate these channels. However, these mechanisms remain unknown and should form the basis of future detailed study.

Beyond mGluR1α and VGCCs, native GABA_B_Rs interact with a variety of transmembrane and cytosolic proteins in when measured in tissue lysates.^28^^,^^29^ If the same interactomes are present in SST INs and whether other associated proteins are similarly lost from these cells remain unexplored. Indeed, the functional association of GABA_B_Rs with other postsynaptic effectors (e.g., Kir3 channels) differ in diverse IN types.^8^^,^^9^^,^^12^ As INs make up only approximately 10% of neurons in the hippocampus,^48^ efforts to identify the GABA_B_R interactome using bulk protein samples^29^ have likely underestimated cell-type-specific effects. Future recent advances in single-cell proteomic analysis^49^ may allow detection of cell-type-specific GABA_B_R binding partners, crucial to determine such detailed function.

### GABA_B_R downregulation from SST IN dendrites impairs hippocampal circuit function

It has long been appreciated that acute GABA_B_R activation leads to paradoxical functions at the circuit level, notably enhanced LTP^50^^,^^51^ and increase seizure activity,^52^ despite directly hyperpolarizing membranes^53^^,^^54^ and inhibiting pre- and postsynaptic Ca^2+^ channels^55^^,^^56^ on PCs. These effects point to a function beyond direct inhibition (favoring a disinhibitory model), exemplified by GABA_B_R inhibition on multiple IN types across the hippocampus.^4^ Our data extends this, providing evidence that prolonged GABA_B_R activation fundamentally shifts the ability of SST INs to undergo LTP on behaviorally relevant timescales. Given that SST IN LTP has been well associated with numerous behavioral functions, including spatial and contextual memory,^24^^,^^40^^,^^57^^,^^58^^,^^59^ this indicates a potential role of GABA_B_Rs in regulating this activity. We confirm that LTP in SST INs is crucial to maintain transfer of information to CA1 via the TA path, as previously reported,^19^ the control of which is impaired when baclofen is pre-applied. This is partially supported by behavioral experiments confirming baclofen administration prior to contextual fear conditioning impairs acquisition and recall of these SST IN-dependent memories (Figure S5). However, given the non-specific actions of systemically administered baclofen on multiple brain structures involved with fear memory,^60^^,^^61^^,^^62^ interpreting these findings is not straightforward. Future studies using CA1 SST IN-specific deletion of GABA_B_ isoforms would help elucidate such effects.

Furthermore, we show that GABA_B_R activation prior to contextual fear conditioning is sufficient to prevent memory encoding. While baclofen has been shown to impair fear conditioning previously,^62^ these studies relied on administering the drug immediately (<15 min) before fear acquisition, suggesting direct inhibitory effects. The pharmacokinetics of baclofen suggests peak brain concentrations with 30 min of IP administration, which remain high for several hours.^63^ Therefore, it is nigh on impossible to estimate when (and if) loss of SST IN LTP supersedes inhibition driven by GABA_B_R inhibition. Future studies investigating GABA_B_R surface localization on SST INs over hours and days post baclofen injection may reveal the temporal resolution of this relationship, but are outside the scope of this project.

### Translational importance

Baclofen is a common and important medicine, primarily prescribed for muscle spasm, but also tested for alcohol dependency, neurodevelopmental disorders, among other conditions (Romito et al.^64^), up to 100 mg/day; but is associated with many common neurological side effects. Thus, understanding how baclofen affects cortical circuits may inform its best use, particularly in conditions where GABAergic inhibition has also been implicated.

One of the most common clinical features associated with baclofen administration is the paradoxical induction of seizure activity, both in patients^65^ and in rodent models.^52^ Originally proposed as a potential anti-seizure medication,^66^ effects of baclofen on hippocampal circuits has previously been described, leading to seizure-like activity.^67^ The baclofen-dependent loss of SST IN excitatory signaling, we observe may favor long-term reduction in local SST IN inhibition, which would be predicted to cause seizures.^68^ Interestingly, we recently showed higher GABA_B_R signaling in PCs in patients, who have experienced seizures.^69^ It is tempting to speculate that such increases may result from reduced inhibitory tone, leading to compensatory upregulation of receptor expression. Further study is required to determine if this is the case.

A recent off-label use for high-dose baclofen has been in treating addiction, particularly alcoholism use disorder.^70^ In rodents, GABA_B_Rs have been shown to be lost in the cortex following chronic alcohol administration^71^ and ventral tegmental area (VTA) following administration of amphetamine or cocaine,^26^ albeit not tested in SST INs. However, it has been shown that acute alcohol administration bidirectionally affects SST INs, with low doses increasing activity and high doses attenuating activity.^72^ SST INs are present in cortical^15^^,^^73^ and VTA^74^ circuits, and their activity is strongly downregulated in chronic alcohol-consuming mice, which may be corrected by inhibiting them directly.^75^ Our data suggest that prolonged baclofen administration may prevent SST IN activity, akin to chemogenetic approaches used previously, thus may reflect a mechanism by which baclofen could produce therapeutic benefit.

### Summary and outlook

Here, we show in hippocampal SST INs that prolonged activation of GABA_B_Rs is sufficient to cause downregulation of the receptor in the plasma membrane through a PP2A-dependent process, which also leads to reduced surface density of mGluR1α and Ca_V_1.2 to prevent synaptic plasticity at CA1 PC inputs. These data provide compelling evidence of how the inhibitory molecule baclofen exerts a paradoxical disinhibitory effect by reducing SST IN function, which has relevance for hippocampal function and disease.

### Limitations of the study

While we show that decrease of surface localized GABA_B_Rs from SST IN dendritic membranes occurs following 20 min of baclofen application, using our approach we were not able to identify the specific temporal dynamics of this loss. If such effects occur rapidly (within seconds) or require longer (minutes to hours) remains unclear. Nevertheless, baclofen as a therapy is often administered at moderate to high doses (up to 100 mg/day) and has a CSF half-life of several hours,^63^ thus such temporal dynamics—while mechanistically interesting—are somewhat negated by these pharmacokinetics. Further studies identifying whether GABA_B_R reduction from SST IN dendrites displays dose- and time-dependence would provide important insight to baclofen’s therapeutic use. Furthermore, we have not identified how long SST INs take to recover full plasticity potential following baclofen administration, which may inform circuit function.

One unusual observation in our data were that OA appeared to occlude labeling of GABA_B1_ in SDS-FRL experiments, both on SST INs and CA1 PCs (Figure S3). We do not have a good explanation for this effect. One possibility is that SDS-FRL relies on immunolabeling of membrane-bound proteins^76^ and OA as a large polypeptide interacts with an epitope on PP2A close to its interaction site with GABA_B1_. Such an interaction may mask or occlude antibody binding that would otherwise not be observed with the use of small molecule pharmacology, such as fostriecin. A similar phenomenon has been proposed for the binding of the amyloid precursor protein (APP) to GABA_B1_, which has been indicated in SDS-FRL to be diminished in APP overexpressing mice,^77^ but in which mice GABA_B_R function is unaltered.^78^ These data indicate the importance of providing multiple lines of evidence to support conclusions based on physiology or anatomy alone, which we have provided through comparative use of OA and fostriecin that both selectively inhibit PP2A.^79^

Finally, our data suggest that in SST INs GABA_B_Rs likely interact with themselves, and influence the function of—mGluR1α, Ca_v_1.2, and PP2A. Such proteins do not appear on previously published interactomes of GABA_B1_ or GABA_B2_ in the hippocampus.^29^ Our data suggest the existence of cell-type-specific interactomes, which may not be detectable at a whole brain, or even region-specific level. Such an endeavor would almost certainly identify cell-type-specific differences even within neurochemically defined classes, as highlighted by previous works.^4^^,^^12^ However, such analyses are beyond the scope of the current study. Future work should seek to identify, which proteins GABA_B_Rs interact with, in a cell-type-specific manner, and potentially if such interactions change with age or neuropathology.

## Resource availability

### Lead contact

Further information and requests for resources and reagents should be directed to and will be fulfilled by the lead contact, Sam A. Booker (sbooker@ed.ac.uk).

### Materials availability

This study did not generate new unique reagents.

### Data and code availability

• This paper does not report original code
• All raw data contributing to this manuscript is provided in supplementary table “Sethumadhavan et al._raw data and statistics.”
• Any additional information required to reanalyze the data reported in this paper is available from the lead contact upon request.

## Acknowledgments

We wish to thank Natalie Wernet and Julia Bank for technical support, Britt Van De Gevel for additional statistical analysis, BVS technicians for supporting animal husbandry, and members of the Booker, Vida, and Kulik labs for discussions of the data. Funding was provided by: 10.13039/501100000265UK Research and Innovation (UKRI) - Medical Research Council (MR/Y014529/1, to S.A.B.); DFG (FOR 2134, to I.V. and A.K.), BIOSS-2 (to A.K.), CRC-TRR 384/1 (to I.V. and A.K.) the Patrick Wild Center (to S.A.B.), and the Simons Initiative for the Developing Brain (SFARI: 529085).

## Author contributions

Conceptualization, S.A.B. and A.K.; methodology, N.S., S.A.B., M.A.W., A.S., D.L., and A.K.; validation, S.A.B., and A.K.; formal analysis, S.A.B., A.K., N.S., M.A.W., and A.S.; investigation, S.A.B., N.S., M.A.W., D.L., A.S., and R.M.L.; writing – original draft, S.A.B.; writing – review and editing, S.A.B., N.S., I.V., and A.K.; supervision, S.A.B., I.V., and A.K.; funding acquisition, S.A.B., I.V., and A.K.

## Declaration of interests

The authors declare no competing interests.

## STAR★Methods

### Key resources table

**Table:** 

REAGENT or RESOURCE	SOURCE	IDENTIFIER
**Antibodies**		

Anti-GABAB1	Kulik et al.^80^	B17
Anti-mGluR1α	Frontier Institute	Af660; RRID: AB_2571801
Anti-Ca_V_1.2	NeuroMab	N263/31; RRID: AB_2877476
Anti-GFP	Aves Labs	GFP-1010; RRID:AB_2307313
Anti-somatostatin	BMA Biomedicals	T-4103; RRID: AB_518614
Streptavidin Alexa Fluor 633	Invitrogen	S21374; RRID: AB_2336066

**Chemicals, peptides, and recombinant proteins**		

R-Baclofen	Abcam Biochemicals	ab120325
CGP-55,845	Abcam Biochemicals	ab120337
Picrotoxin	Abcam Biochemicals	ab120315
CNQX	Abcam Biochemicals	ab120044
DL-AP-5	Abcam Biochemicals	ab120271
QX-314	Abcam Biochemicals	ab120118
S- DPHG	Abcam Biochemicals	ab120007
Nifedipine	Abcam Biochemicals	ab120135
LY-367,385	HelloBio	HB0398
Okadaic Acid	HelloBio	HB0468
Fostriecin	HelloBio	HB0293

**Deposited data**		

Raw data	This study	Supplementary Materials

**Experimental models: Organisms/strains**		

Mouse: vGAT-venus	Wang et al.^81^	N/A
Mouse: Somatostatin-cre (Ssttm2.1(cre)Zjh/J)	Jackson Laboratory	013044
Mouse: Ai32^RCL−ChR2(H134R)/EYFP^	Jackson Laboratory	024109
Mouse: C57/Bl6J (CRL substrain)	Charles River	027

**Software and algorithms**		

pClamp	Molecular Devices	
Stimfit	Guzman et at.^82^	
FIJI/ImageJ	Schindelin et al.^83^	
WinLTP 3.01	WinLTP Ltd.	
RStudio	Posit Software, PBC	
Graphpad Prism 11	Dotmatics	

### Experimental model and study participant details

All *ex vivo* electrophysiology was performed in brain slices prepared from male and female mice (2-4 months old), with all procedures performed according to Home Office (ASPA, 2013) and The University of Edinburgh Ethical Board (AWERB), under project licence PP2262369. Mice were maintained on a C57/Bl6J^CRL^ background and expressed yellow-fluorescent protein (YFP) under the vesicular GABA transporter (vGAT).^81^ Mice were housed on a 12h light/dark cycle, with *ad libitum* access to food and water.

For SDS-FRL, male mice (8-week-old, *n* = 12) either wild-type or in which SST-INs selectively expressed Channelrhodopsin2(ChR2)-YFP fusion protein were derived from crossing SST-Cre and Ai32^RCL-ChR2(H134R)/EYFP^ transgenic mice. For behavioural analysis, male C57/Bl6J mice (8–12 weeks old, *n* = 6 per group) were used. Care and handling of the animals prior to and during the experimental procedures followed European Union and national regulations (German Animal Welfare Act) and all experiments were performed in accordance with institutional guidelines (University of Freiburg, Germany) with permission from local authority (Freiburg, X21/04B).

### Method details

#### Acute brain slice preparation

Brain slices were prepared as previously described.^84^ Mice were terminally anaesthetised with isoflurane, decapitated, then their brains rapidly dissected into ice-cold sucrose-modified artificial cerebrospinal fluid (sucrose-ACSF; ACSF; in mM: 87 NaCl, 2.5 KCl, 25 NaHCO_3_, 1.25 NaH_2_PO_4_, 25 glucose, 75 sucrose, 7 MgCl_2_, 0.5 CaCl_2_) which was saturated with carbogen (95% O_2_/5% CO_2_). Brains were glued to a vibratome stage (Leica VT1200S, Leica, Germany), then 300 μm (for whole-cell recordings) or 500 μm (for extracellular recordings) horizontal slices containing the hippocampi were prepared. Following slicing, brain slices were placed in either: a submerged holding chamber containing sucrose-ACSF warmed to 35 °C for 30 min, then at room temperature; or on small squares of filter paper placed in a liquid/gas interface chamber, containing recording ACSF (in mM: 125 NaCl, 2.5 KCl, 25 NaHCO_3_, 1.25 NaH_2_PO_4_, 25 glucose, 1 MgCl_2_, 2 CaCl_2_) and bubbled with carbogen.

#### Whole-cell patch clamp recordings

Briefly, 400 μm horizontal hippocampal slices were prepared from 60-90 day-old C57/Bl6J mice as previously described.^84^ For recording, slices were transferred to a submerged chamber maintained at 31 ± 1°C. Whole-cell recordings were made using pipettes filled with either K-gluconate or Cs-gluconate based solutions. Voltage-clamp recordings were performed from a potential of either -65 mV or -60 mV, while current-clamp recordings were made from resting membrane potential. Intrinsic properties of recorded neurons were characterized on their voltage response to hyper- to depolarizing current steps, in current-clamp. HVA calcium transients, mGluR EPSCs, and GABA_B_R-mediated currents were measured in the presence of 10 μM CNQX, 50 μM DL-AP-5, and 50 μM picrotoxin. GABA_A_R IPSCs were elicited by placing a bipolar stimulating electrode in *str. lacunosum-moleculare* approximately 200-300 μm rostral to the recorded neuron, in the presence of 10 μM CNQX, 50 μM DL-AP-5. LTP was induced at alveus inputs to SST INs with EPSCs elicited by a bipolar stimulating electrode placed in the alveus, in the presence of 50 μM picrotoxin. Following baseline recording, associative LTP was induced by a theta-burst stimulation paradigm^36^ – consisting of 5 trains of 5 electrical stimuli (100 Hz) paired with a depolarization to -20 mV, which was repeated 3 times at 30 second intervals. EPSCs were measured for 25 minutes following induction. For all stimulation, the pulse duration was 200 μs delivered via a constant voltage generator. For HVA VGCC activation, once a whole-cell patch-clamp recording was obtained using a Cs-Gluconate internal solution (containing QX-314), the cell was allowed to dialyse for 5 minutes. Then at -60 mV voltage-clamp, 3 families of depolarizing steps were applied (-60 to 20 mV, 5 mV steps, 500 ms duration). The L-type VGCC blocker nifedipine (20 μM) was then applied to the bath for 3 minutes and 3 further families of depolarizing steps obtained. All traces were then P/N subtracted based on the 5 mV step and the peak current measured. The peak current under nifedipine was then subtracted from control. For mGluR EPSC experiments, slow-EPSCs were elicited by focal puff application of the Group 1 mGluR agonist DPHG (100 μM, 500 ms, 20 psi, diluted from stock in 150 mM NaCl) delivered via a patch pipette placed ∼50 μm from the cell body. The specificity of the mGluR current was assessed by subsequent bath application of the mGluR1α-specific antagonist LY-367,385 (100 μM).

For pre-application experiments, slices were transferred to a holding chamber containing recording ACSF and 20 μM R-baclofen for 20 minutes, slices were then placed in the recording chamber and rinsed with ACSF without R-baclofen for 4-6 minutes prior to recording. To block PP2A activity, slices were transferred to a chamber containing ACSF and 1 μM Okadaic Acid or 50 nM Fostriecin for at least 1 hour prior to recording or baclofen pre-application. All other drugs were applied directly to the perfusing ACSF.

#### Field recordings

For field excitatory postsynaptic potential (fEPSP) recordings, hippocampal slices were transferred to an interface recording chamber perfused with carbogenated artificial cerebrospinal fluid (ACSF) at 2–3 mL/min and maintained at 30 ± 1 °C. Recording electrodes (1–3 MΩ) were pulled from borosilicate glass capillaries (1.5 mm outer/0.86 mm inner diameter; Harvard Apparatus, UK) using a horizontal puller and filled with recording ACSF. Slices were visualised with a wide-field microscope (Leica, Germany), and electrodes were positioned in the *str. L-M* of CA1.

Extracellular fEPSPs were evoked using a paired-pulse protocol delivered via a bipolar stimulating electrode placed in *str. L-M*, approximately 500 μm to 1 mm from the recording electrode, to activate the temporoammonic pathway. A second stimulating electrode was positioned in parallel within the alveus of CA1. To activate GABA_B_R, slices were perfused with 20 μM baclofen for 20 min, followed by a 20-min washout period.

A 10-min baseline was recorded under control conditions or following baclofen washout. LTP was first induced via alveus stimulation using a high-frequency stimulation (HFS) protocol consisting of three 100 Hz trains of 20 pulses delivered 30 s apart, or a protocol consisting of five 100 Hz trains of 5 pulses, delivered 30 s apart. LTP was then induced in str. L-M using an HFS protocol of 1x 100 Hz train of 100 pulses delivered 30 s apart.

Potentiation was monitored for 60 min following induction. LTP magnitude was quantified as the mean fEPSP slope measured 50–60 min post-induction, normalised to the 10-min baseline. LTP was considered successful if the 50–60 min slope exceeded baseline by >10%. Recordings were excluded if baseline stability, assessed by comparing mean slopes at 1–2 min and 9–10 min, varied ±10%. Signals were filtered online (1 Hz high-pass, 500 Hz low-pass) and digitised at 10 kHz. Data were acquired and analysed offline using WinLTP (v3.01, University of Bristol, UK).

#### Neuronal visualization and immunohistochemistry

All neurons were filled with biocytin during recording, fixed overnight in 4% PFA and labelled with streptavidin and antibodies to SST according to previous methods.^85^ Slices were washed in PBS then blocked for 1 hour at room temperature (10% NGS, 0.3% Triton X-100 and 0.05% NaN_3_ in PBS). Following blocking slices were incubated with primary antibodies at 4°C for 48h (1:1000 rabbit SST-14, T-4103, BMA Biomedicals, Switzerland; 5% NGS, 0.3% Triton X-100 and 0.05% NaN_3_ in PBS), washed in PBS then transferred to a secondary antibody solution containing fluorescent-conjugated streptavidin (1:500 goat anti-rabbit AlexaFluor 488, 1:500 Streptavidin AlexaFluor 633, 3% NGS, 0.1% Triton X-100, 0.05% NaN_3_). Following labeling the slices were washed in 0.1M PB and mounted on glass slides with Vectashield HardSet mounting medium (Vector Labs, UK). To identify SST Ins Confocal image stacks of recorded neurons were taken on a Leica SP8 confocal microscope using a 63x (1.4 NA) objective at 1024 × 1024 resolution (Z step size 1 μm).

#### Electron microscopy

##### Acute slice preparation and pharmacology for SDS-FRL

Acute hippocampal slices were prepared as previously described.^84^ Animals were sedated with isoflurane then they were decapitated and the brains were rapidly dissected and chilled in semi-frozen carbogenated (95% O_2_/5% CO_2_) sucrose-substituted artificial cerebrospinal fluid (sucrose-ACSF, in mM: 87 NaCl, 2.5 KCl, 25 NaHCO_3_, 1.25 NaH_2_PO_4_, 25 glucose, 75 sucrose, 7 MgCl_2_, 0.5 CaCl_2_, 1 Na-pyruvate, 1 Na-ascorbate). Transverse hippocampal slices (200 μm thick) were cut on a vibratome (VT1200s, Leica, Germany) and stored submerged in sucrose-ACSF warmed to 35°C for at least 30 min and subsequently at room temperature (RT). Both solutions were equilibrated with 95% O_2_ and 5% CO_2_ gas mixture throughout experiments. Acute slices were divided into four groups for pharmacological treatment: 1, incubated in ACSF (control, 30 min), 2, ACSF + GABA_B_R agonist baclofen (Bac, 20 μM, 20 min), 3, ACSF + protein phosphatase 2A inhibitor fostriecin (FS, 50 nM, 90 min), 4, ACSF + fostriecin + baclofen (FS/Bac, FS 50 nM 90 min + Bac 20 μM, 20 min). After pharmacological manipulations, slices were transferred into fixative containing 1% paraformaldehyde and 15% saturated picric acid made up in 0.1 M phosphate buffer (PB) overnight (O/N) at 4 °C.

##### SDS-FRL immunoelectron microscopy

After fixation, slices were cryoprotected in 30% glycerol in 0.1M PB O/N at 4°C.^10^ Blocks containing str. O/A were trimmed from the slices and frozen under high-pressure (HPM 100, Leica). The frozen samples were fractured at -140°C and the fractured faces were coated with carbon, (5 nm), platinum-carbon (2 nm) and carbon (18 nm) in a freeze-fracture replica machine (ACE 900, Leica). Replicas were digested at 80°C in a solution containing 2.5% SDS and 20% sucrose diluted in 15 mM Tris buffer (TB, pH 8.3) for 20 hr. Subsequently, replicas were washed in washing buffer containing 0.05% bovine serum albumin (BSA, Roth, Germany) and 0.1% Tween 20 in 50 mM Tris-buffered saline (TBS) and then blocked in a solution containing 5% BSA and 0.1% Tween 20 in TBS for 1 hr at RT. Afterwards, replicas prepared from SST-cre mice were incubated in the following mixtures of primary antibodies in a solution containing 1% BSA and 0.1% Tween 20 made up in TBS for 3 days at 15°C: (i) GABA_B1_ (B17, rabbit, 10 μg/ml^80^) and green fluorescence protein (GFP-1010, chicken 0.4 μg/ml, Aves Labs, Oregon; Booker et al., 2020) or (ii) Ca_v_1.2 (mouse, 50 μg/ml, NeuroMab Facility, California; Booker et al., 2018) and GFP. Replicas were washed then reacted with 6 nm (GABA_B1_ and Ca_v_1.2) and 18 nm (GFP) gold particle-conjugated secondary antibodies (1:30, Jackson ImmunoResearch Europe, Cambridgeshire) O/N at 15°C. The replicas prepared from WT mice were incubated in Ca_v_1.2 (mouse, 50 μg/ml, NeuroMab Facility, California), GABA_B1_ (B17, rabbit, 10 μg/ml) and metabotropic glutamate receptor 1α-subunit (mGluR1α, Guinea pig, 0.4 μg/ml, Frontier Institute, Hokkaido; Booker et al., 2018) in a solution containing 1% BSA and 0.1% Tween 20 made up in TBS for 3 days at 15°C. Replicas were washed then reacted with 5 nm (GABA_B1_), 10 nm (Ca_v_1.2), and 18 nm (mGluR1α) gold particle-conjugated secondary antibodies (1:30, Jackson ImmunoResearch Europe, Cambridgeshire) O/N at 15°C. Finally, replicas were washed in TBS then distilled water and mounted on 100-mesh grids.

##### Electron microscopy and quantitative analysis of protein density

Labeled replicas were analyzed under an electron microscope (JEM 2100 Plus). Since all antibodies target intracellular protein epitopes, immunoreactivity can be detected on the plasma membrane’s protoplasmic face (P-face) but not the exoplasmic face (E-face). The surface density of receptors and ion channels was determined by dividing the absolute number of particles labeling molecules of interest by the surface of the respective segments of the dendritic shafts of GFP-positive SST-INs on replicas.

##### Behavioural analysis

To investigate the role of GABA_B_R activation in fear learning, we performed a fear conditioning experiment. Male C57/Bl6J mice (8–12 weeks old, *n* = 6 per group) were handled and habituated. On the day of the test, mice were given a two-minute baseline period before receiving three-foot shocks (0.06 mA) spaced one minute apart. Following the last shock, the mice remained in the fear conditioning arena for an additional minute, then they were transferred to the home cage. The entire training session lasted for approx. five minutes. Mice were divided into test and control groups. Test group were injected with baclofen (i.p., 2 mg/kg), while the control group received a vehicle (0.9% NaCl). One hour following the baclofen or vehicle injection, freezing time was measured in the same context as recall in order to access fear memory.

### Quantification and statistical analysis

For *ex vivo* electrophysiology, one or two cells or slices were recorded per animal and treatment, limiting the ability to assess intra-animal variability. As such cells and slices were treated as independent replicates and statistical analyses were conducted on these data. Parametric and non-parametric tests were applied as appropriate, including two-way ANOVA, Student’s t-test, Mann–Whitney U-tests, and Wilcoxon signed-rank tests. For SDS-FRL, where many dendritic profiles were sampled per animal replicate, we performed linear-mixed effects modelling (LMM) of using the “lmer” function to limit the potential inclusion of pseudoreplication. Animal was assigned as random effects, then type III ANOVA (Satterthwaites method) was performed on the model to assess main effects (e.g. drug treatment). If a statistical interaction between main effects was observed, then post-hoc analysis was performed (Tukey post hoc tests). Key statistical test results are shown on figures and in the text. For all data is shown as box-plots with median and 25–75% percentiles displayed in the box; minimum and maximum extents are shown as whiskers, with individual data points shown from cell or animal averages. For timecourses and current-voltage plots, data is shown as mean ± SEM. No data was excluded from analysis. Statistical significance was defined as *p* < 0.05. Statistical tests and graphing were performed using either GraphPad Prism (GraphPad Software v10.4.1, San Diego, CA, United States) or R-Studio.

## Supplemental information

Document S1. Figures S1–S5 and Tables S1 and S2

Data S1. A summary of all data collected, analyzed, and presented in the figures of this article

## References

[1] Bartos M., Vida I., Jonas P. (2007). Synaptic mechanisms of synchronized gamma oscillations in inhibitory interneuron networks. Nat. Rev. Neurosci..

[2] Burgess N., O'Keefe J. (1996). Neuronal computations underlying the firing of place cells and their role in navigation. Hippocampus.

[3] Feldman D.E. (2012). The spike-timing dependence of plasticity. Neuron.

[4] Kulik Á., Booker S.A., Vida I. (2018). Differential distribution and function of GABABRs in somato-dendritic and axonal compartments of principal cells and interneurons in cortical circuits. Neuropharmacology.

[5] Guet-McCreight A., Skinner F.K., Topolnik L. (2020). Common principles in functional organization of VIP/calretinin cell-driven disinhibitory circuits across cortical areas. Front. Neural Circuits.

[6] Williams L.E., Holtmaat A. (2019). Higher-order thalamocortical inputs gate synaptic long-term potentiation via disinhibition. Neuron.

[7] Booker S.A., Althof D., Degro C.E., Watanabe M., Kulik Á., Vida I. (2017). Differential surface density and modulatory effects of presynaptic GABA_B_ receptors in hippocampal cholecystokinin and parvalbumin basket cells. Brain Struct. Funct..

[8] Booker S.A., Althof D., Gross A., Loreth D., Müller J., Unger A., Fakler B., Varro A., Watanabe M., Gassmann M. (2017). KCTD12 auxiliary proteins modulate kinetics of GABAB receptor-mediated inhibition in cholecystokinin-containing interneurons. Cereb. Cortex.

[9] Booker S.A., Gross A., Althof D., Shigemoto R., Bettler B., Frotscher M., Hearing M., Wickman K., Watanabe M., Kulik Á., Vida I. (2013). Differential GABAB-receptor-mediated effects in perisomatic-and dendrite-targeting parvalbumin interneurons. J. Neurosci..

[10] Booker S.A., Harada H., Elgueta C., Bank J., Bartos M., Kulik A., Vida I. (2020). Presynaptic GABAB receptors functionally uncouple somatostatin interneurons from the active hippocampal network. eLife.

[11] Booker S.A., Loreth D., Gee A.L., Watanabe M., Kind P.C., Wyllie D.J.A., Kulik Á., Vida I. (2018). Postsynaptic GABABRs inhibit L-type calcium channels and abolish long-term potentiation in hippocampal somatostatin interneurons. Cell Rep..

[12] Degro C.E., Vida I., Booker S.A. (2024). Postsynaptic GABAB-receptor mediated currents in diverse dentate gyrus interneuron types. Hippocampus.

[13] Mott D.D., Li Q., Okazaki M.M., Turner D.A., Lewis D.V. (1999). GABAB-receptor–mediated currents in interneurons of the dentate-hilus border. J. Neurophysiol..

[14] Price C.J., Scott R., Rusakov D.A., Capogna M. (2008). GABAB receptor modulation of feedforward inhibition through hippocampal neurogliaform cells. J. Neurosci..

[15] Booker S.A., Vida I. (2018). Morphological diversity and connectivity of hippocampal interneurons. Cell Tissue Res..

[16] Ali A.B., Thomson A.M. (1998). Facilitating pyramid to horizontal oriens-alveus interneurone inputs: dual intracellular recordings in slices of rat hippocampus. J. Physiol..

[17] Lacaille J.-C., Mueller A.L., Kunkel D.D., Schwartzkroin P.A. (1987). Local circuit interactions between oriens/alveus interneurons and CA1 pyramidal cells in hippocampal slices: electrophysiology and morphology. J. Neurosci..

[18] Müller C., Remy S. (2014). Dendritic inhibition mediated by O-LM and bistratified interneurons in the hippocampus. Front. Synaptic Neurosci..

[19] Yanovsky Y., Sergeeva O.A., Freund T.F., Haas H.L. (1997). Activation of interneurons at the stratum oriens/alveus border suppresses excitatory transmission to apical dendrites in the CA1 area of the mouse hippocampus. Neuroscience.

[20] Lovett-Barron M., Turi G.F., Kaifosh P., Lee P.H., Bolze F., Sun X.H., Nicoud J.F., Zemelman B.V., Sternson S.M., Losonczy A. (2012). Regulation of neuronal input transformations by tunable dendritic inhibition. Nat. Neurosci..

[21] Singh S., Topolnik L. (2023). Inhibitory circuits in fear memory and fear-related disorders. Front. Neural Circuits.

[22] Leão R.N., Mikulovic S., Leão K.E., Munguba H., Gezelius H., Enjin A., Patra K., Eriksson A., Loew L.M., Tort A.B.L., Kullander K. (2012). OLM interneurons differentially modulate CA3 and entorhinal inputs to hippocampal CA1 neurons. Nat. Neurosci..

[23] Udakis M., Pedrosa V., Chamberlain S.E.L., Clopath C., Mellor J.R. (2020). Interneuron-specific plasticity at parvalbumin and somatostatin inhibitory synapses onto CA1 pyramidal neurons shapes hippocampal output. Nat. Commun..

[24] Udakis M., Claydon M.D.B., Zhu H.W., Oakes E.C., Mellor J.R. (2025). Hippocampal OLM interneurons regulate CA1 place cell plasticity and remapping. Nat. Commun..

[25] Dinamarca M.C., Raveh A., Schneider A., Fritzius T., Früh S., Rem P.D., Stawarski M., Lalanne T., Turecek R., Choo M. (2019). Complex formation of APP with GABAB receptors links axonal trafficking to amyloidogenic processing. Nat. Commun..

[26] Padgett C.L., Lalive A.L., Tan K.R., Terunuma M., Munoz M.B., Pangalos M.N., Martínez-Hernández J., Watanabe M., Moss S.J., Luján R. (2012). Methamphetamine-evoked depression of GABAB receptor signaling in GABA neurons of the VTA. Neuron.

[27] Raveh A., Turecek R., Bettler B. (2015). Mechanisms of fast desensitization of GABA(B) receptor-gated currents. Adv. Pharmacol..

[28] Schwenk J., Metz M., Zolles G., Turecek R., Fritzius T., Bildl W., Tarusawa E., Kulik A., Unger A., Ivankova K. (2010). Native GABAB receptors are heteromultimers with a family of auxiliary subunits. Nature.

[29] Schwenk J., Pérez-Garci E., Schneider A., Kollewe A., Gauthier-Kemper A., Fritzius T., Raveh A., Dinamarca M.C., Hanuschkin A., Bildl W. (2016). Modular composition and dynamics of native GABAB receptors identified by high-resolution proteomics. Nat. Neurosci..

[30] Hearing M., Kotecki L., Marron Fernandez de Velasco E., Fajardo-Serrano A., Chung H.J., Luján R., Wickman K. (2013). Repeated cocaine weakens GABAB-Girk signaling in layer 5/6 pyramidal neurons in the prelimbic cortex. Neuron.

[31] Li X., Terunuma M., Deeb T.G., Wiseman S., Pangalos M.N., Nairn A.C., Moss S.J., Slesinger P.A. (2020). Direct interaction of PP2A phosphatase with GABAB receptors alters functional signaling. J. Neurosci..

[32] Hirono M., Yoshioka T., Konishi S. (2001). GABAB receptor activation enhances mGluR-mediated responses at cerebellar excitatory synapses. Nat. Neurosci..

[33] Tabata T., Araishi K., Hashimoto K., Hashimotodani Y., van der Putten H., Bettler B., Kano M. (2004). Ca2+ activity at GABAB receptors constitutively promotes metabotropic glutamate signaling in the absence of GABA. Proc. Natl. Acad. Sci. USA.

[34] Baude A., Nusser Z., Roberts J.D., Mulvihill E., McIlhinney R.A., Somogyi P. (1993). The metabotropic glutamate receptor (mGluRlα) is concentrated at perisynaptic membrane of neuronal subpopulations as detected by immunogold reaction. Neuron.

[35] Blasco-Ibáñez J.M., Freund T.F. (1995). Synaptic input of horizontal interneurons in stratum oriens of the hippocampal CA1 subfield: structural basis of feed-back activation. Eur. J. Neurosci..

[36] Topolnik L., Azzi M., Morin F., Kougioumoutzakis A., Lacaille J.C. (2006). mGluR1/5 subtype-specific calcium signalling and induction of long-term potentiation in rat hippocampal oriens/alveus interneurones. J. Physiol..

[37] Vasuta C., Artinian J., Laplante I., Hébert-Seropian S., Elayoubi K., Lacaille J.C. (2015). Metaplastic regulation of CA1 Schaffer collateral pathway plasticity by Hebbian MGluR1a-mediated plasticity at excitatory synapses onto somatostatin-expressing interneurons. eNeuro.

[38] Terunuma M., Vargas K.J., Wilkins M.E., Ramírez O.A., Jaureguiberry-Bravo M., Pangalos M.N., Smart T.G., Moss S.J., Couve A. (2010). Prolonged activation of NMDA receptors promotes dephosphorylation and alters postendocytic sorting of GABAB receptors. Proc. Natl. Acad. Sci. USA.

[39] Matos M., Bosson A., Riebe I., Reynell C., Vallée J., Laplante I., Panatier A., Robitaille R., Lacaille J.C. (2018). Astrocytes detect and upregulate transmission at inhibitory synapses of somatostatin interneurons onto pyramidal cells. Nat. Commun..

[40] Honoré E., Lacaille J.-C. (2022). Object location learning in mice requires hippocampal somatostatin interneuron activity and is facilitated by mTORC1-mediated long-term potentiation of their excitatory synapses. Mol. Brain.

[41] Shen C., Ding H., Zhang S., Xu C., Zou B., Ji S., Liu Y.R., Li Y., Zhou R., Liang J. (2026). Functional and structural basis of a negative allostery within GABAB hetero-tetramers. Nat. Commun..

[42] Benke D., Blackburn T.P. (2010).

[43] Couve A., Thomas P., Calver A.R., Hirst W.D., Pangalos M.N., Walsh F.S., Smart T.G., Moss S.J. (2002). Cyclic AMP–dependent protein kinase phosphorylation facilitates GABAB receptor–effector coupling. Nat. Neurosci..

[44] Pin J.-P., Bettler B. (2016). Organization and functions of mGlu and GABAB receptor complexes. Nature.

[45] Sistiaga A., Sánchez-Prieto J. (2000). Protein phosphatase 1 and 2A inhibitors prolong the switch in the control of glutamate release by group I metabotropic glutamate receptors: characterization of the inhibitory pathway. J. Neurochem..

[46] Lecca S., Pelosi A., Tchenio A., Moutkine I., Lujan R., Hervé D., Mameli M. (2016). Rescue of GABAB and GIRK function in the lateral habenula by protein phosphatase 2A inhibition ameliorates depression-like phenotypes in mice. Nat. Med..

[47] Minobe E., Maeda S., Xu J., Hao L., Kameyama A., Kameyama M. (2014). A new phosphorylation site in cardiac L-type Ca2+ channels (Cav1. 2) responsible for its cAMP-mediated modulation. Am. J. Physiol. Cell Physiol..

[48] Freund T.F., Buzsáki G. (1996). Interneurons of the hippocampus. Hippocampus.

[49] Karlsson F., Simonetti M., Galonska C., Karlsson M., van Ooijen H., Kallas T., Thiagrajan D., Schweitzer M., Larsson L., Tajvar P. (2025). Single-cell protein interactomes by the proximity network assay. Blood.

[50] Davies C.H., Starkey S.J., Pozza M.F., Collingridge G.L. (1991). GABAB autoreceptors regulate the induction of LTP. Nature.

[51] Mott D.D., Lewis D.V. (1991). Facilitation of the induction of long-term potentiation by GABAB receptors. Science.

[52] Mott D.D., Bragdon A.C., Lewis D.V., Wilson W.A. (1989). Baclofen has a proepileptic effect in the rat dentate gyrus. J. Pharmacol. Exp. Ther..

[53] Lüscher C., Jan L.Y., Stoffel M., Malenka R.C., Nicoll R.A. (1997). G protein-coupled inwardly rectifying K+ channels (GIRKs) mediate postsynaptic but not presynaptic transmitter actions in hippocampal neurons. Neuron.

[54] Sodickson D.L., Bean B.P. (1996). GABAB receptor-activated inwardly rectifying potassium current in dissociated hippocampal CA3 neurons. J. Neurosci..

[55] Chalifoux J.R., Carter A.G. (2011). GABAB receptor modulation of voltage-sensitive calcium channels in spines and dendrites. J. Neurosci..

[56] Thompson S.M., Gähwiler B.H. (1992). Comparison of the actions of baclofen at pre-and postsynaptic receptors in the rat hippocampus in vitro. J. Physiol..

[57] Grigoryan G., Harada H., Knobloch-Bollmann H.S., Kilias A., Kaufhold D., Kulik A., Eyre M.D., Bartos M. (2023). Synaptic plasticity at the dentate gyrus granule cell to somatostatin-expressing interneuron synapses supports object location memory. Proc. Natl. Acad. Sci. USA.

[58] Hainmueller T., Cazala A., Huang L.-W., Bartos M. (2024). Subfield-specific interneuron circuits govern the hippocampal response to novelty in male mice. Nat. Commun..

[59] Yuan M., Cazala A., Goedeke S., Leibold C., Sauer J.F., Bartos M. (2025). Predictive goal coding by dentate gyrus somatostatin-expressing interneurons in male mice. Nat. Commun..

[60] Castellano C., Cestari V., Cabib S., Puglisi-Allegra S. (1993). Strain-dependent effects of post-training GABA receptor agonists and antagonists on memory storage in mice. Psychopharmacology.

[61] Delaney A.J., Crane J.W., Holmes N.M., Fam J., Westbrook R.F. (2018). Baclofen acts in the central amygdala to reduce synaptic transmission and impair context fear conditioning. Sci. Rep..

[62] Heaney C.F., Bolton M.M., Murtishaw A.S., Sabbagh J.J., Magcalas C.M., Kinney J.W. (2012). Baclofen administration alters fear extinction and GABAergic protein levels. Neurobiol. Learn. Mem..

[63] Balerio G.N., Rubio M.C. (2002). Pharmacokinetic-pharmacodynamic modeling of the antinociceptive effect of baclofen in mice. Eur. J. Drug Metab. Pharmacokinet..

[64] Romito J.W., Turner E.R., Rosener J.A., Coldiron L., Udipi A., Nohrn L., Tausiani J., Romito B.T. (2021). Baclofen therapeutics, toxicity, and withdrawal: A narrative review. SAGE Open Med..

[65] Kofler M., Kronenberg M.F., Rifici C., Saltuari L., Bauer G. (1994). Epileptic seizures associated with intrathecal baclofen application. Neurology.

[66] Ault B., Nadler J.V. (1983). Anticonvulsant-like actions of baclofen in the rat hippocampal slice. Br. J. Pharmacol..

[67] Dugladze T., Maziashvili N., Börgers C., Gurgenidze S., Häussler U., Winkelmann A., Haas C.A., Meier J.C., Vida I., Kopell N.J., Gloveli T. (2013). GABA(B) autoreceptor-mediated cell type-specific reduction of inhibition in epileptic mice. Proc. Natl. Acad. Sci. USA.

[68] Drexel M., Rahimi S., Sperk G. (2022). Silencing of Hippocampal Somatostatin Interneurons Induces Recurrent Spontaneous Limbic Seizures in Mice. Neuroscience.

[69] Wilson M.A., Sumera A., Taylor L.W., Meftah S., McGeachan R.I., Modebadze T., Jayasekera B.A.P., Cowie C.J.A., LeBeau F.E.N., Liaquat I. (2025). Phylogenetic divergence of GABAB receptor signaling in neocortical networks over adult life. Nat. Commun..

[70] de Beaurepaire R., Sinclair J.M.A., Heydtmann M., Addolorato G., Aubin H.J., Beraha E.M., Caputo F., Chick J.D., de La Selle P., Franchitto N. (2019). The Use of Baclofen as a Treatment for Alcohol Use Disorder: A Clinical Practice Perspective. Front. Psychiatry.

[71] Peng S.-Y., Shi Z., Zhou D.S., Wang X.Y., Li X.X., Liu X.L., Wang W.D., Lin G.N., Pan B.X., Voon V. (2021). Reduced motor cortex GABABR function following chronic alcohol exposure. Mol. Psychiatry.

[72] Li M., Cabrera-Garcia D., Salling M.C., Au E., Yang G., Harrison N.L. (2021). Alcohol reduces the activity of somatostatin interneurons in the mouse prefrontal cortex: A neural basis for its disinhibitory effect?. Neuropharmacology.

[73] Rudy B., Fishell G., Lee S., Hjerling-Leffler J. (2011). Three groups of interneurons account for nearly 100% of neocortical GABAergic neurons. Dev. Neurobiol..

[74] Nagaeva E., Zubarev I., Bengtsson Gonzales C., Forss M., Nikouei K., de Miguel E., Elsilä L., Linden A.M., Hjerling-Leffler J., Augustine G.J., Korpi E.R. (2020). Heterogeneous somatostatin-expressing neuron population in mouse ventral tegmental area. eLife.

[75] Dao N.C., Brockway D.F., Suresh Nair M., Sicher A.R., Crowley N.A. (2021). Somatostatin neurons control an alcohol binge drinking prelimbic microcircuit in mice. Neuropsychopharmacology.

[76] Masugi-Tokita M., Shigemoto R. (2007). High-resolution quantitative visualization of glutamate and GABA receptors at central synapses. Curr. Opin. Neurobiol..

[77] Martín-Belmonte A., Aguado C., Alfaro-Ruiz R., Moreno-Martínez A.E., de la Ossa L., Aso E., Gómez-Acero L., Shigemoto R., Fukazawa Y., Ciruela F., Luján R. (2022). Nanoscale alterations in GABAB receptors and GIRK channel organization on the hippocampus of APP/PS1 mice. Alz. Res. Therapy.

[78] Meftah S., Wilson M.A., Elliott J., McLay L., Dobrovolskis V., Rosencrans S., Taylor L.W., Mugnaini C., Mostallino R., Durrant C.S., Booker S.A. (2026). GABAB Receptor signaling in CA1 Pyramidal Cells is not Regulated by Aging in the APP/PS1 Mouse Model of Amyloid Pathology. eNeuro.

[79] Neale D.A., Morris J.C., Verrills N.M., Ammit A.J. (2025). Understanding the regulatory landscape of protein phosphatase 2A (PP2A): Pharmacological modulators and potential therapeutics. Pharmacol. Ther..

[80] Kulik Á., Nakadate K., Nyíri G., Notomi T., Malitschek B., Bettler B., Shigemoto R. (2002). Distinct localization of GABAB receptors relative to synaptic sites in the rat cerebellum and ventrobasal thalamus. Eur. J. Neurosci..

[81] Wang Y., Kakizaki T., Sakagami H., Saito K., Ebihara S., Kato M., Hirabayashi M., Saito Y., Furuya N., Yanagawa Y. (2009). Fluorescent labeling of both GABAergic and glycinergic neurons in vesicular GABA transporter (VGAT)–Venus transgenic mouse. Neuroscience.

[82] Guzman S.J., Schlögl A., Schmidt-Hieber C. (2014). Stimfit: quantifying electrophysiological data with Python. Front Neuroinform..

[83] Schindelin J., Arganda-Carreras I., Frise E., Kaynig V., Longair M., Pietzsch T., Preibisch S., Rueden C., Saalfeld S., Schmid B. (2012). Fiji: an open-source platform for biological-image analysis. Nat. Methods.

[84] Oliveira L.S., Sumera A., Booker S.A. (2021). Repeated whole-cell patch-clamp recording from CA1 pyramidal cells in rodent hippocampal slices followed by axon initial segment labeling. STAR Protoc..

[85] Yang Y., Booker S.A., Clegg J.M., Quintana-Urzainqui I., Sumera A., Kozic Z., Dando O., Martin Lorenzo S., Herault Y., Kind P.C. (2023). Identifying foetal forebrain interneurons as a target for monogenic autism risk factors and the polygenic 16p11. 2 microdeletion. BMC Neurosci..

