# Supplementary material for: PP2A-dependent downregulation of GABAB receptors modulates somatostatin interneurons function and plasticity
Source: iScience. 2026 Oct 27;29(11):117878. doi: 10.1016/j.isci.2026.117878 (PMC13733333; doi:10.1016/j.isci.2026.117878)
Supplement: Supplementary file 1 [file mmc1.pdf]

iScience, Volume *29*

Supplemental information

PP2A-dependent downregulation of $GABA_B$ receptors modulates somatostatin interneurons function and plasticity

Nithya Sethumadhavan, Max A. Wilson, Anna Sumera, Desiree Loreth, Rita M. Loureiro, Imre Vida, Akos Kulik, and Sam A. Booker

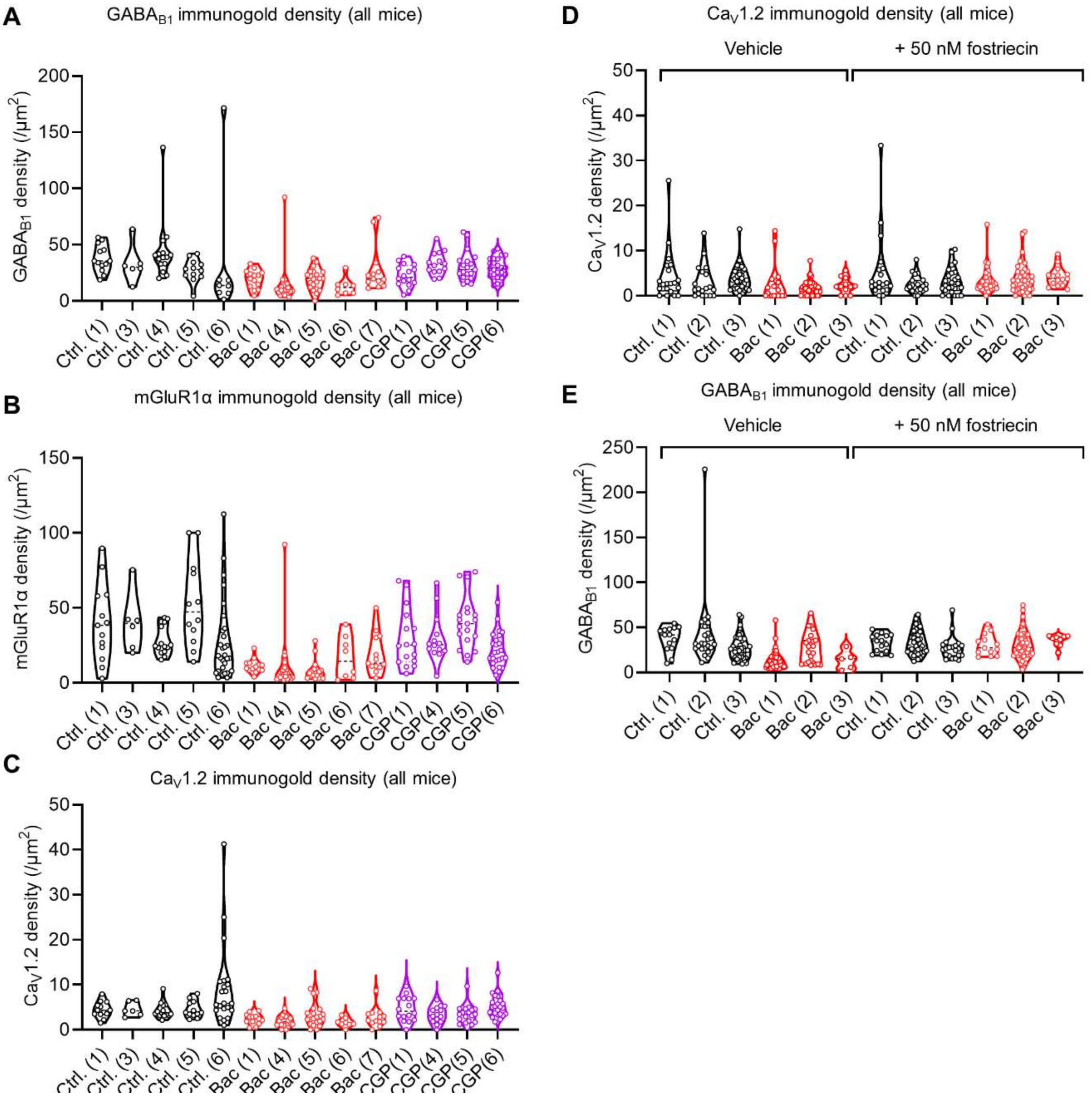

Figure S1: Summary of all immunogold density measurements from all dendritic profiles measured in each biological replicate for the current study, related to Figure 1. **A**) mGluR1α density in from SDS-FRIL replicas from slices treated with vehicle control (black), 20 minutes of 20 µM baclofen (red) or 5 µM CGP-55,845 (purple). **B**) the same data, but for $Ca_V1.2$ labelling. **C**) the same data but for GABAB1 labelling. **D**) $Ca_V1.2$ immunogold labelling on dendrites in SDS-FRIL replicas from mice expressing SST-ChR2 for control (black) and baclofen (red) treated slices. **E**) the same data as D but for GABAB immunogold labelling. All data is shown as individual density for each dendritic profile, with violin plots for each animal.

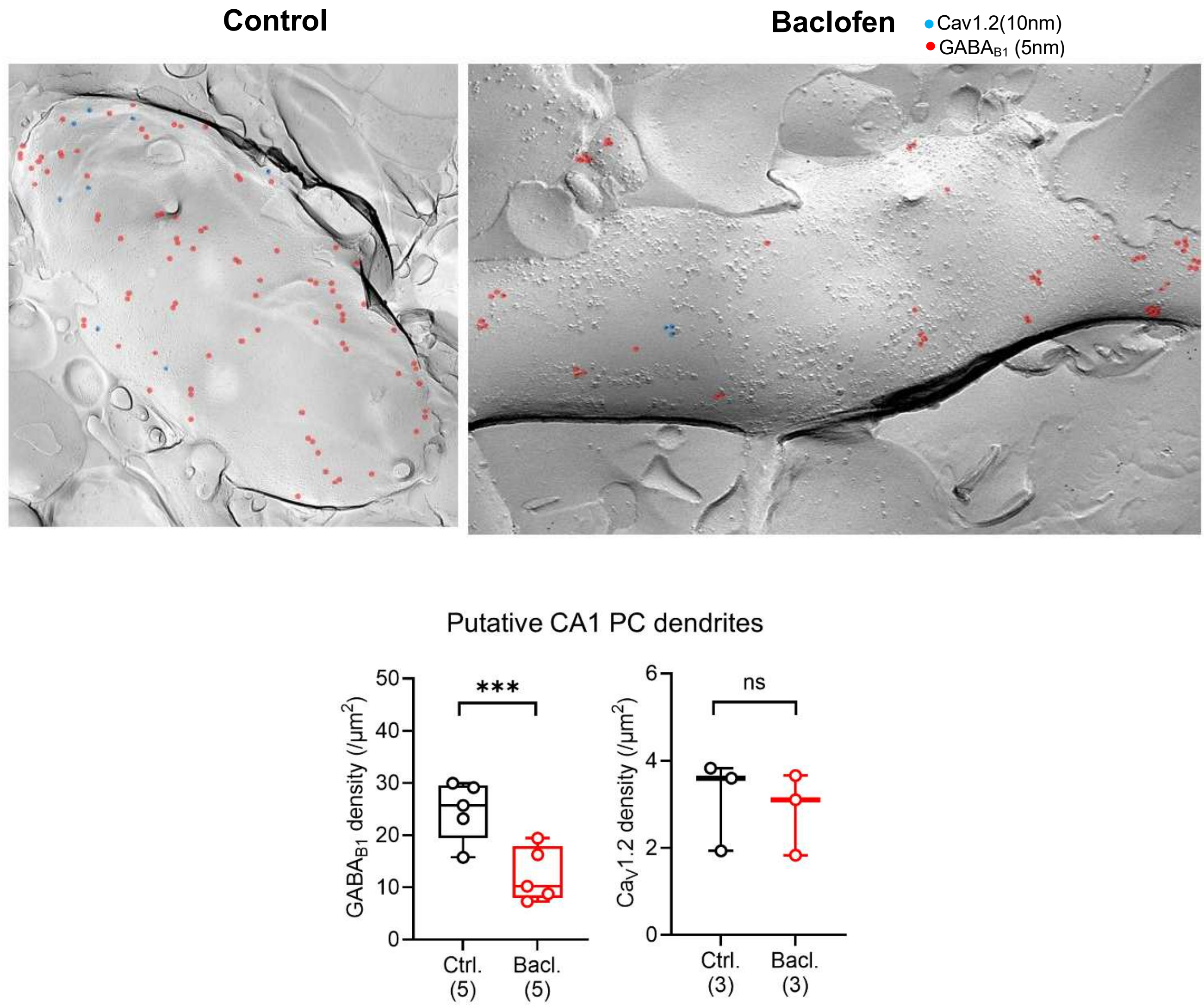

Figure S2: Immunogold labelling for $GABA_{B1}$ and $Ca_V1.2$ on putative PC dendrites identified in the same replicas, related to Figure 1. Putative CA1 PC dendrites were identified on the basis of possessing dendritic spines a prominent postsynaptic density. Data is shown from control (black) and baclofen pre-treated (red) replicas from 3 mice, and depicted as box-plots overlaid by individual animal averages. Statistics shown from LMM analysis, ns – $p>0.05$, *** - $p<0.0001$.

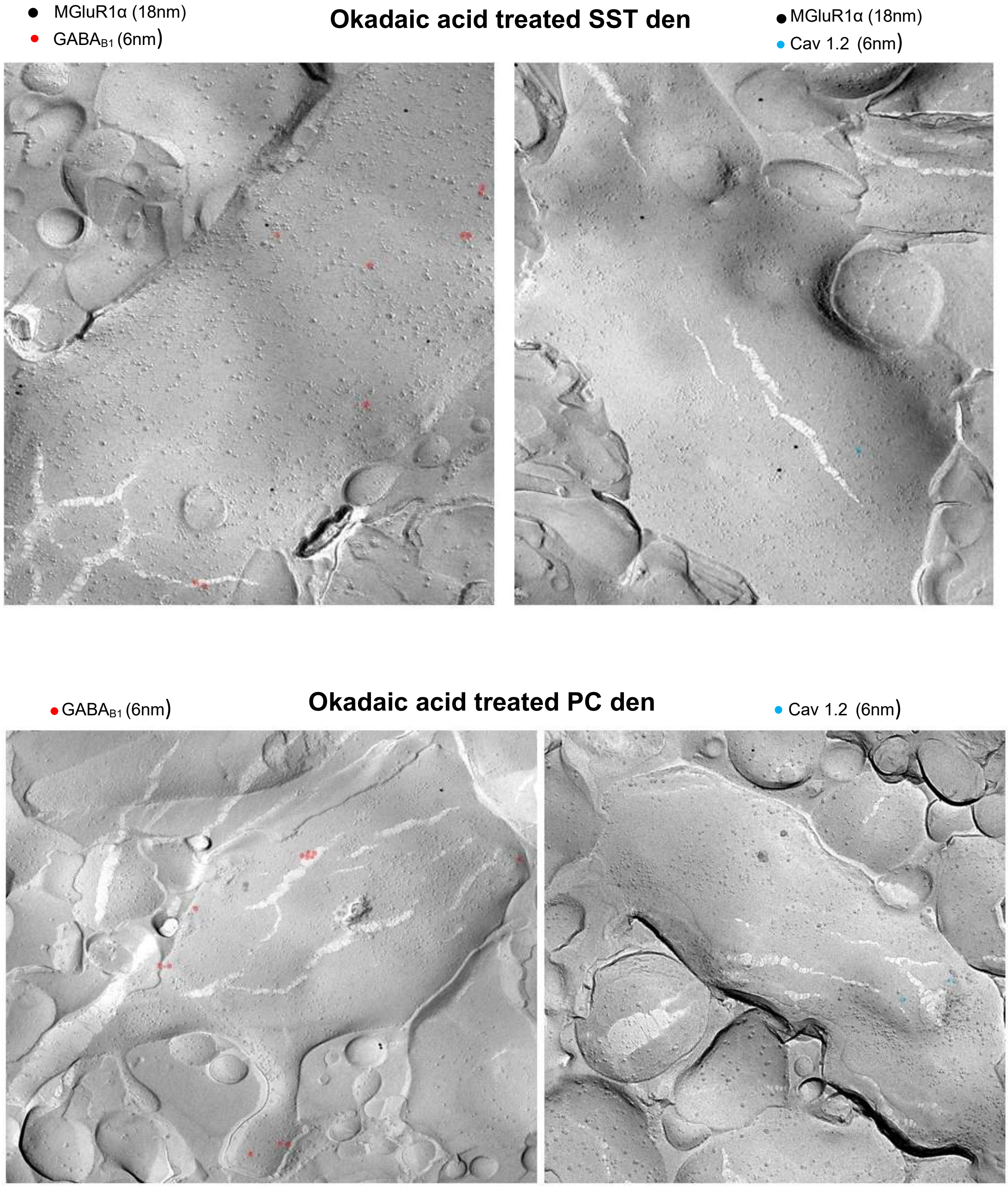

Figure S3: Okadaic acid prevents occludes $GABA_{B1}$ immunolabelling in SDS-FRL, *, related to Figure 3.* Example images from a putative SST IN (top) and CA1 PC (bottom) labelled for mGluR1α (18 nm immunogold), GABAB1 (red highlight, 6 nm) and CaV1.2 (blue highlight, 6 nm). Note the abundance of strong immunogold labelling under any condition.

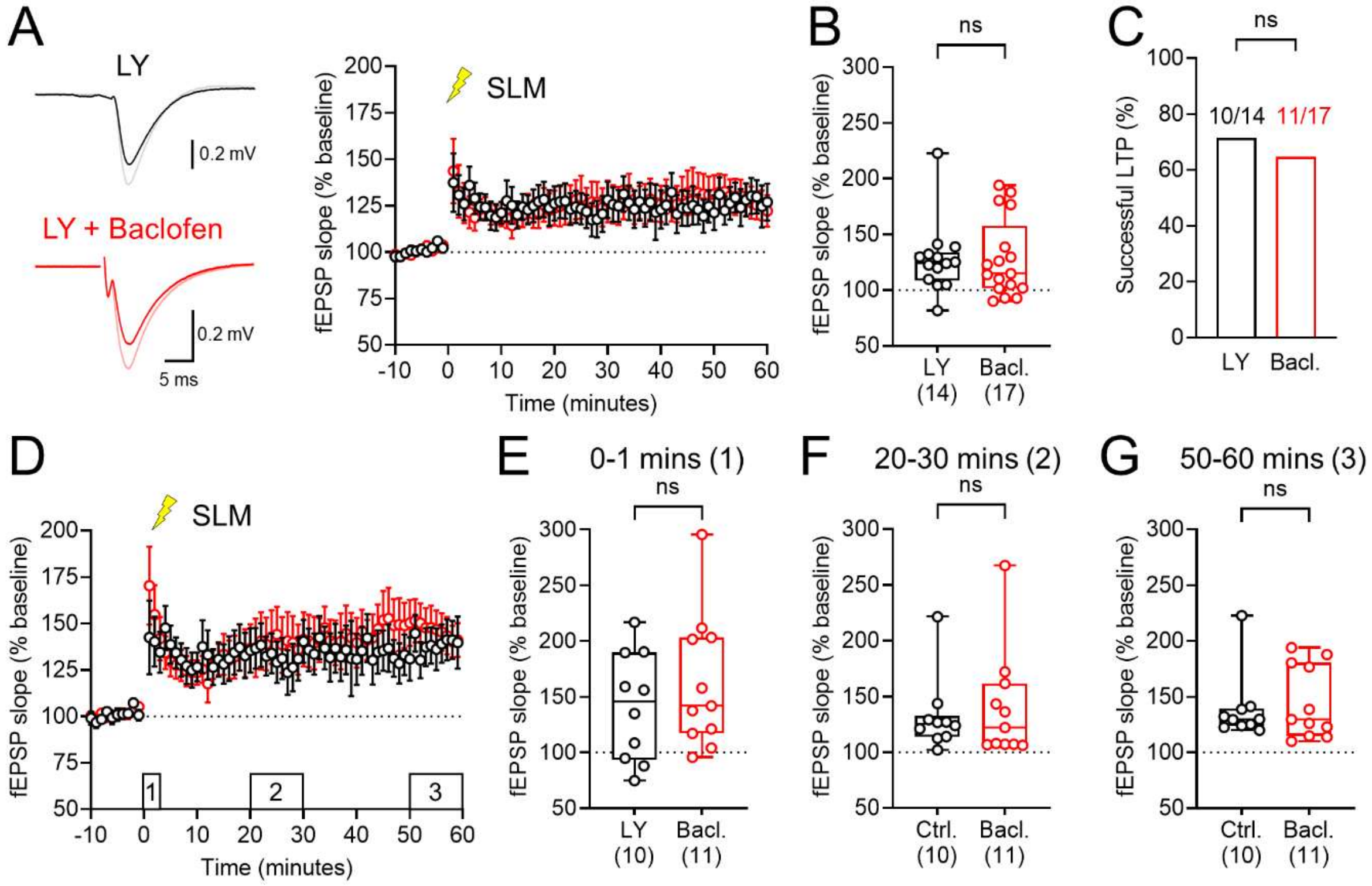

Figure S4: Pharmacological inhibition of SST IN plasticity with mGluR1α antagonists prevents $GABA_BR$ internalisation induced facilitation of temporoammonic LTP, *related to Figure 7*. A) Example fEPSP traces recorded in *str. L-M* in response to temporoammonic stimulation, and following induction of LTP in the *alveus* in the presence of LY-367,385 (LY) to antagonise mGluR1α in control (black) and 20 μM baclofen pre-treated slices (red). Right, time-course of LTP in *str. L-M* in both slice conditions, recorded in the presence of LY. **B**) Quantification of the magnitude of LTP in all recordings performed in LY. **C**) Proportion of recordings in both control and baclofen pre-treatment that successfully induced LTP (>10% facilitation at 50-60 minutes post HFS) in the presence of LY. **D**) Time-course of successful LTP recordings induced in *str. L-M* in the presence of LY. Quantification of fEPSP facilitation in control and baclofen pre-treated slices at 0-1 (**E**), 20-30 (**F**), and 50-60 minutes (**G**) post *str. L-M* HFS, all in the presence of LY. Data is shown as either mean ± SEM (**A, D**), box-plots showing 25-75% box with median, with maximum range (**B, E, F, G**), pr proportions (**C**). Individual cell data is shown overlaid. Statistics shown as: ns – p>0.05, from Mann Whitney non-parametric tests or Chi-squared test (**C**).

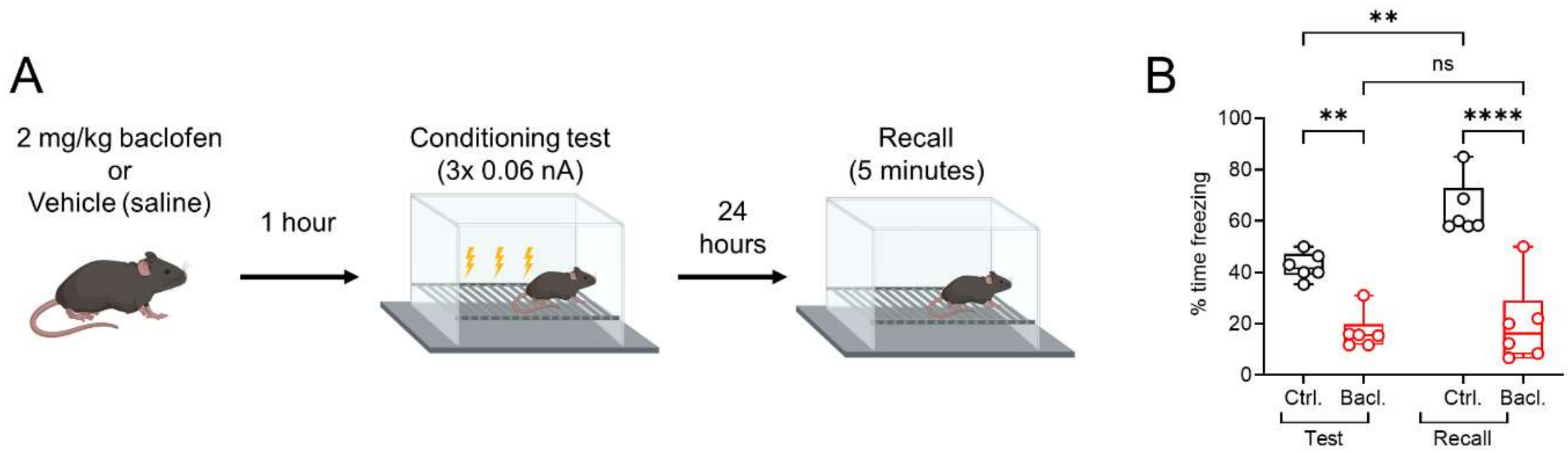

Figure S5: Prior GABA$_B$*R activation impairs contextual memory formation, related to Figure 7.* A Schematic of contextual fear conditioning experiments, in which mice were dosed with 2 mg/kg baclofen 1 hour prior to training. B Quantification of % time freezing during the test and recall phases of conditional fear conditioning. Data is shown as box-plots showing 25-75% box with median, with maximum range. Individual cell data is shown overlaid. Statistics shown as: ns – p>0.05, ** - p<0.01, **** - p<0.0001; from 2-way ANOVA with Holm-Sidak post-tests.

Electrophysiological Property	Control (18 cells, 14 mice)	Baclofen pre-application (14 cells, 8 mice)	P value
Membrane potential (mV)	-60.5 ± 8.8	-59.1 ± 6.7	0.638
Input resistance (MΩ)	327.7 ± 118.6	218.2 ± 128.6	**0.018 (*)**
Membrane time-constant (ms)	29.9 ± 13.9	20.2 ± 12.8	0.051
Capacitance (pF)	93.7 ± 37.9	95.8 ± 42.4	0.884
Rheobase (pA)	40.0 ± 32.5	106.3 ± 69.2	**0.003 (**)**
Voltage threshold (mV)	-41.6 ± 6.1	-38.4 ± 6.5	0.162
AP amplitude (mV)	87.3 ± 16.6	74.8 ± 15.5	**0.0378 (*)**
AP 20-80% rise-time (ms)	0.15 ± 0.06	0.16 ± 0.07	0.865
AP half-height width (ms)	0.49 ± 0.17	0.47 ± 0.17	0.751
AP maximum rise ($mV.ms^{-1}$)	370.3 ± 162.0	294.5 ± 124.9	0.158
AP maximum decay ($mV.ms^{-1}$)	185.1 ± 84.2	169.7 ± 66.2	0.580
Sag amplitude (mV)	6.6 ± 4.1	4.4 ± 3.7	0.126
Sag amplitude % of max	19.0 ± 9.3	16.4 ± 13.0	0.514
Adaptation index	1.8 ± 0.8	1.4 ± 0.3	0.166
FI Slope ($AP. pA^{-1}$)	0.19 ± 0.05	0.11 ± 0.06	**0.007 (**)**

Table S1: Basal electrophysiological parameters of identified SST INs under control conditions and following baclofen pre-treatment, *related to Figure 5.* Data are shown as mean ± SD for key electrophysiological parameters measured from hyper to depolarising current steps (-125 to +125 pA, 25 pA steps, 500 ms duration). Statistics shown as output from Student's unpaired t-tests.

Electrophysiological Property	Control (12 cells, 10 mice)	Baclofen pre-application (7 cells, 5 mice)	P value
Membrane potential (mV)	-64.4 ± 7.2	-68.2 ± 8.5	0.312
Input resistance (MΩ)	106.4 ± 43.1	109.2 ± 34.2	0.887
Membrane time-constant (ms)	18.8 ± 4.7	10.3 ± 2.5	**0.0004 (***)**
Capacitance (pF)	199.2 ± 81.2	106.3 ± 51.4	**0.015 (*)**
Rheobase (pA)	200.0 ± 104.5	228.6 ± 95.1	0.561
Voltage threshold (mV)	-40.4 ± 4.8	-40.7 ± 4.0	0.878
AP amplitude (mV)	108.9 ± 11.9	111.1 ± 9.5	0.673
AP 20-80% rise-time (ms)	0.14 ± 0.03	0.14 ± 0.01	0.873
AP half-height width (ms)	0.86 ± 0.14	0.73 ± 0.05	**0.032 (*)**
AP maximum rise (mV.ms^{-1})	461.2 ± 117.4	426.2 ± 59.2	0.475
AP maximum decay (mV.ms^{-1})	92.6 ± 17.4	117.2 ± 8.6	**0.003 (**)**
Sag amplitude (mV)	6.0 ± 3.2	7.1 ± 3.8	0.491
Sag amplitude % of max	14.2 ± 4.9	15.9 ± 5.7	0.508
Adaptation index	4.65 ± 1.67	6.1 ± 3.6	0.262
FI Slope (AP. pA^{-1})	0.16 ± 0.05	0.14 ± 0.05	0.546

Table S2: Basal electrophysiological parameters of CA1 PCs under control conditions and following baclofen pre-treatment, *related to Figure 5.* Data are shown as mean ± SD for key electrophysiological parameters measured from hyper to depolarising current steps (-500 to +500 pA, 100 pA steps, 500 ms duration). Statistics shown as output from Student's unpaired t-tests.
